# Multipass Target Search in Natural Environments

**DOI:** 10.3390/s17112514

**Published:** 2017-11-02

**Authors:** Michael J. Kuhlman, Michael W. Otte, Donald Sofge, Satyandra K. Gupta

**Affiliations:** 1 Institute for Systems Research, Department of Mechanical Engineering, University of Maryland, College Park, MD 20742, USA; 2 National Research Council RAP Postdoctoral Associate at Naval Research Laboratory, Washington, DC 20375, USA; michael.otte.ctr@nrl.navy.mil; 3 Navy Center for Applied Research in Artificial Intelligence, Naval Research Laboratory, Washington, DC 20375, USA; donald.sofge@nrl.navy.mil; 4 Center for Advanced Manufacturing, Aerospace and Mechanical Engineering Department, University of Southern California, Los Angeles, CA 90089, USA; guptask@usc.edu

**Keywords:** path planning, information gathering, branch and bound, target search, coverage planning

## Abstract

Consider a disaster scenario where search and rescue workers must search difficult to access buildings during an earthquake or flood. Often, finding survivors a few hours sooner results in a dramatic increase in saved lives, suggesting the use of drones for expedient rescue operations. Entropy can be used to quantify the generation and resolution of uncertainty. When searching for targets, maximizing mutual information of future sensor observations will minimize expected target location uncertainty by minimizing the entropy of the future estimate. Motion planning for multi-target autonomous search requires planning over an area with an imperfect sensor and may require multiple passes, which is hindered by the submodularity property of mutual information. Further, mission duration constraints must be handled accordingly, requiring consideration of the vehicle’s dynamics to generate feasible trajectories and must plan trajectories spanning the entire mission duration, something which most information gathering algorithms are incapable of doing. If unanticipated changes occur in an uncertain environment, new plans must be generated quickly. In addition, planning multipass trajectories requires evaluating path dependent rewards, requiring planning in the space of all previously selected actions, compounding the problem. We present an anytime algorithm for autonomous multipass target search in natural environments. The algorithm is capable of generating long duration dynamically feasible multipass coverage plans that maximize mutual information using a variety of techniques such as ϵ-admissible heuristics to speed up the search. To the authors’ knowledge this is the first attempt at efficiently solving multipass target search problems of such long duration. The proposed algorithm is based on best first branch and bound and is benchmarked against state of the art algorithms adapted to the problem in natural Simplex environments, gathering the most information in the given search time.

## 1. Introduction

Earthquakes and similar disasters in remote areas pose a challenge for relief efforts when the transportation infrastructure is damaged. Victims in such disasters have a greater chance of survival the sooner they are discovered; conducting an extensive search even just a few hours faster will save many more lives. Survival rates of earthquake victims continually drop significantly the longer the delay before rescue, with a sharp drop off at around 48 h [1,2]. In the 2013 earthquake that hit Lushan, China, Chinese researchers provided an autonomous rotary-wing UAV to assist search and rescue efforts [2]. Interest in using drones for search and rescue and assessing dangerous situations has also been increasing in local fire fighting departments [3].

Consider a scenario where an earthquake or flood causes a building to collapse, trapping survivors who are difficult to access by ground. It would be advantageous to have aerial vehicles autonomously fly at low altitudes navigating over rubble searching for survivors. However, such environments may be subject to change, requiring quick decision making while acting on a previously generated plan. There are several reasons why generating a multipass coverage plan is advantageous for target search. Scenarios involving noisy sensors or targets that appear or disappear over time require multiple passes to correctly determine whether or not a target is present. Further, given constraints in mission duration, the agent must be able to properly decide where to search, and for how long, before returning to base.

Mutual information [4] is an ideal metric to maximize for improving search. Mutual information measures the dependence of two random variables, such as a noisy sensor measurement in the future and the current best estimate of a target’s location. Picking a future sensor measurement that maximizes mutual information will effectively reduce the expected uncertainty of the target’s location [5]. Most importantly, assessing the mutual information of an action allows one to formally assess the trade-offs between searching a region multiple times or searching another region not searched, making it an ideal metric for multipass multi-target search [6]. The mutual information of conditionally independent events has been shown to be submodular [6], which implies that multiple correlated sensor measurements offer diminishing returns in how much information is gathered when summing the total information of each measurement individually. When trying to maximize the submodular reward function of mutual information, the reward is *path dependent* requiring one to plan in the space of trajectories (Section 3), making planning more computationally burdensome.

This presents several challenges to which we will outline our proposed solution. The algorithm must be able to react quickly to new information that can affect the search plan, suggesting an anytime formulation. An anytime algorithm is an algorithm that quickly finds a feasible solution, and continually improves the solution as available planning time permits. Greedy approaches to informative path planning that only consider all immediate actions are quick to react to changes in the environment, but may get trapped [7,8]. However, previous work has shown that for submodular optimization problems, greedy approaches can get to within a constant factor of the optimal solution [6,9]. Similarly, model predictive control based techniques can look multiple time steps into the future [10] further improving performance, but can be trapped similarly. While both discrete [11] and sample based motion planners [12] have been used in information gathering problems and are capable of generating plans of long duration, they have only been applied to relatively simple environments and have not been used to generate multipass coverage plans, with the exception of the authors’ previous work in the area [13].

We present what we believe to be the first multipass coverage planner that maximizes mutual information for long duration trajectories that span the entire mission duration. Since such path planning problems are at least as hard as other NP-hard planning problems, use of admissible heuristics to guide the search is essential to improve algorithm performance. While there are many admissible heuristics for path planning, these do not extend well to multipass coverage due to the path dependence of the reward. We discuss the heuristics we developed for multipass coverage planning and demonstrate how they improve solution quality in the given computational time budget. We also extend three other state-of-the-art coverage planners to handle the multipass coverage planning problem and benchmark how the four algorithms perform in simulated natural environments based on total information gathered, expected time for human ground crews to search a cell after the autonomous search, and the time to compute the solution.

This paper significantly extends the preliminary work by the authors [13] by benchmarking the proposed ϵ-admissible branch and bound algorithm in realistic simulated environments, and extending the analysis of the algorithm’s performance. The new realistic environment models account for sensors viewing multiple cells, the physics of the vehicle, and realistic variations in region shapes using state of the art environment decomposition techniques on simulated obstacle occupancy grids generated by gradient noise. Experimental results compare the performance of the proposed ϵ-admissible branch and bound algorithm to other state of the art algorithms and also assesses how well in expectation the various algorithms guide human relief crews using a simplified effort allocation model. Findings show that ϵ-admissible branch and bound is able to gather the most information and reduces the expected time for ground crews to search a cell the most. Such quality plans come at the cost of requiring the most time to compute, however. We conclude our analysis with a discussion on the low cost of supercomputing capabilities contrasted with the large value of a statistical life.

**Figure 1:**
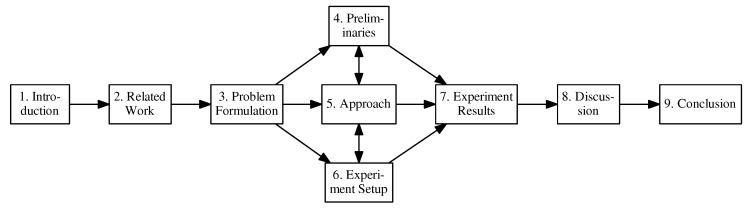
Road-map of the organization of the paper.

The paper is organized as follows, following the road-map in Figure 1. Section 1 offers an introduction and motivation to the topics presented. Section 2 covers related work in the literature. Section 3 offers a formal problem formulation for multipass coverage planning for target search. The detailed description of the algorithm presented in this paper is divided into three related sections. Cross references are provided to establish ties between related concepts; readers may skip the cross references on an initial read. Section 4 introduces important preliminary concepts and introduces the benchmark algorithms. Section 5 outlines the proposed algorithm in the paper, and outlines how the analysis will be conducted. Section 6 discusses implementation details of the algorithm outlining the simulated natural environment developed for benchmarking the algorithms. Section 7 covers the experiments and their results, Section 8 discusses the experiment results and Section 9 concludes the work and discusses future work.

## 2. Related Work

Research interest in probabilistic target search appeared as early as 1956 [14] studying the probability of detection of a target given various sensor conditions. Since then, a large body of work has been developed on the task of target search, including many surveys of the subject [15,16,17].

First and foremost, our literature survey will focus on problems requiring active search where agents keep moving to locate targets [18,19] instead of static surveillance [20] (consider placement of static sentries in the art gallery problem to detect intruders [21]). Several important factors that delineate different approaches in the literature include the number of targets to be tracked, the assumptions about the target motion model, and who is searching for the targets. Some approaches are only for finding a single target, while others are for finding multiple targets. When working under a Bayesian framework, it is common to track a probability density function of a single target’s location [22]. This can be extended to a predefined number of targets in parallel [23,24], or using random finite sets to track the likelihood of the existence of a random number of targets [7,18,25].

Further, whether or not targets are stationary or dynamic affects the search. If targets are known to be stationary (or static), one could divide the search space into cells on a grid and track hypotheses in each cell [7,22,26]. Or, one can track the number of targets contained within each cell [27,28]. Since our work assumes stationary targets, there is a grid of cells, but the approach collects multiple cells into regions for faster evaluation of coverage plans by planning at the coarser granularity of searching regions instead of cells, requiring fewer actions for a full duration coverage plan at the cost of generating suboptimal plans. Targets that react and move according to the agent’s position can be assumed to have an adversarial relationship characterized by pursuit-evasion games [16]. Alternatively, target motion can be modeled as a Markov process which is unaffected by the agent’s actions [19,29,30]. Certain approaches also factor coordination between agents to find the target(s), either implicitly or explicitly [30]. Regarding multiagent algorithms, in particular explicit coordination methods are exponentially complex with the number of agents [30]. Multiagent coordination where the agents share and fuse observed data is common [19,23,24,31,32,33]. Multiagent search can also be adversarial where two competing teams are searching for the same target [34].

The use of information theory to improve the gathering of information in target search is widespread. Mutual information is a natural choice for modeling uncertainty reduction, and it works for continuous, discrete, and hybrid representations and is compatible with Bayesian search techniques. However, computing plans of arbitrary length that maximize mutual information is exponentially complex [6]. Many approaches either greedily maximize mutual information [7,8] or look only several actions into the future, similar to model predictive control [10,19,22,23].

In contrast to finding trajectories that maximize mutual information, one could plan trajectories that minimize the time to detect the target [35,36,37]. Minimizing expected time to detection vs. guaranteeing capture or reducing target location uncertainty will be appropriate in different scenarios [37]. However, the mentioned works are formulated for either a single target [35,36] or for a fixed number of targets [37], which is unsuitable for widespread disasters on land where the number of survivors is unknown *a priori*.

Hierarchical approaches can be used to improve the representation of the target distribution or of the environment to speed up the search. Quad-tree like structures representing the target distribution have been used [7,38]. Alternate approaches take advantage of the structure of the environment (e.g., rooms in an indoor map) [30,39] but assume that regions are convex and a line of sight sensor is used. They do not generate coverage plans for each region, which is something we explicitly do. Other work considers uncertain or changing environments [8,40] and focuses on potentially hostile environments that can destroy robots.

Branch and bound is a discrete optimization technique that uses admissible heuristics to guide and significantly speed up the search for the optimal solution compared to e.g., brute force enumeration of all candidate solutions. Branch and bound was initially introduced for target search when search effort of a particular region was indivisible [29].

It is important to note that the term *heuristic* in the robotics community has multiple meanings that vary widely given context, often causing confusion. In the most general sense, heuristics use incomplete information about a problem to find a solution quicker. Certain heuristic algorithms tend to discover suboptimal solutions very quickly (which we denote as suboptimal heuristic algorithms), while admissible heuristics, when combined with a search algorithm such as Branch and Bound or A*, have formal properties that guarantee the discovery of optimal solutions or discover optimal solutions with a minimal number of iterations. Further, inadmissible heuristics do not formally qualify as admissible heuristics but tend to offer performance tradeoffs for the algorithm designer when substituted for admissible heuristics. An ϵ-admissible heuristic offers computational speedups at bounded loss in solution optimality.

Of particular interest is related work that considers a moving target but seeks to maximize the probability of target detection subject to fuel and risk constraints [41]. For information gathering, branch and bound has been used for feature subset selection [42], and has been used in the similar traveling salesman problem (TSP) [43,44]. Branch and bound has been combined with Gaussian Processes for informative path planning [11] and is arguably most closely related to our research. However, the bounding heuristic used cannot account for multiple sensor measurements in the same location, for which we must explicitly account.

An example recursive greedy algorithm heuristic solver solves the submodular orienteering problem [45]. This algorithm, however, assumes that each state is only visited once, making it unsuitable for our application.

In the scenario with uninformative prior information, there is a close resemblance between trajectories that maximize mutual information and coverage plans. A recent survey outlines the various techniques used in coverage planning [46]. Two approximate solutions are of interest which focus on the 2D case. Zelinsky’s algorithm uses dynamic programming to generate a value function, then traverses level sets to cover the entire region [47]. The proposed algorithm can get stuck in the presence of obstacles and must back out to continue its coverage plan. Boustrophedon coverage outlines an approach to partition polygonal environments and suggests using a modified version of depth first search (DFS) to dictate the search order of the various regions [48]. For optimal coverage on graphs, this is equivalent to the traveling salesman problem, which is NP-hard. Interestingly, the solution to the Chinese Postman Problem [49] where the goal is to find the optimal tour covering all edges of a graph can be solved in polynomial time.

With regards to information gathering algorithms not directed towards target search, a time-varying information objective was explored in [12], when using sampling based motion planning for environmental information gathering.

## 3. Problem Formulation

We define a common benchmark problem to solve multipass coverage planning problems by defining an environment (sensor model plus motion model) with an objective function to maximize. We are most interested in (1) the quality of the solution the algorithm generates (how much information the algorithm gathers which in turn changes how long it takes for relief crews to search a cell for survivors) and (2) how long it takes to compute the solution.

Define $W\subset R^{2}$ to be workspace of the robot where w=(x,y)∈W. Let $C=W\times \dot{W}\times \ldots \times T$ be the configuration space consisting of time domain *T*, workspace *W* and all higher order tangent spaces required to define the robot’s trajectory. We assume that the robot starts at point $c_{\mathrm{start}}\in C$. For our application, we are interested in planning in the space of *feasible* trajectories. A feasible trajectory T is a time indexed curve in *C* that starts at $c_{\mathrm{start}}$ and that the robot’s dynamics is capable of reproducing. We denote the space of all feasible trajectories in time domain s∈[0,t] with end time *t* as $T_{t}$. Let $T_{t}={⋃}_{s\in [0,t]}\left\{T_{s}\right\}$ be the space of all valid trajectories whose end times vary between 0 and *t*.

Sensor measurements used for target search are stored in a discrete evidence grid E covering workspace *W*. Instead of counting the sensor measurements the agent observes, we count the sensor measurements observed in each cell separately with k=0,1,2,…. Define grid cell $g_{x,y}=\left[x,x+r)\times [y,y+r\right)$ for $x\in \{x_{min},x_{min}+1,\ldots ,x_{max}-1,x_{max}\}\subseteq N$ and $y\in \{y_{min},y_{min}+1,\ldots ,y_{max}-1,y_{max}\}\subseteq N$ for user defined resolution *r* (typically, r=1) where $W={⋃}_{x,y}g_{x,y}$ such that the collection $g_{x,y}$ exactly covers the workspace.

For each cell g∈E define *X* to be the hidden state of whether or not the cell contains a target. *X* may be discrete or continuous. $Z_{k}\in \left\{0,1\right\}$ is the *k*th sensor measurement in *g* and $Z_{1:k}$ is the collection of the *k* sensor measurements $Z_{1},\ldots ,Z_{k}$. A continuous example is the Bernoulli process where X∼Beta(α,β) and $Z_{k}∼Bernoulli\left(X\right)$. This can model targets that disappear and reappear from view randomly. Let $z_{1:k}$ be an observed sequence of measurements (a realization of $Z_{1:k}$). When it is necessary to denote the particular grid cell in which sensor measurements are taken we shall use superscripts, e.g., $z_{1:k}^{x,y}$ is the observed sequence of measurements in $g_{x,y}$. Each cell in the evidence grid is assumed to be independent from all other cells, that is $X^{i,j}⊥X^{x,y}$ for i≠x and j≠y. For all x,y,k we assume that $X^{x,y}$ is independent of time and thus $Z_{k}^{x,y}$ and $z_{1:k}^{x,y}$ are not affected by the time(s) at which the measurements are taken. The rationale behind this is that targets with restricted mobility (can only move within one grid cell) can be accurately modeled with a stationary process model, plus removing the dependence on time improves the ability to precompute the mutual information of future sensor measurements. Given the forward sensor model $P(Z_{k}=z_{k}|X)$, we compute the inverse sensor model using a recursive application of Bayes’ Rule, (Equation (1)):

(1) $$P\left(X|z_{1:k}\right)=\frac{P\left(Z_{k}=z_{k}|X,z_{1:k-1}\right)P\left(X|z_{1:k-1}\right)}{P(Z_{k}=z_{k}|z_{1:k-1})}$$

in which all sensor measurements are conditionally independent. From this model it is possible to compute the mutual information $I(Z_{k+1};X|z_{1:k})$. In practice, this can be computed *a priori* for any process and sensor model, and tabulated on the number of positive and negative sensor measurements observed (see Section 5 and Table 1 in Section 6.3 ). This model can account for either static (consider the occupancy grid model) or stationary targets (target’s presence at any moment of time is the outcome of a Bernoulli Process, but the target’s distribution is independent of time). The outlined approach in this paper would need to be modified to be used with nonstationary targets (whose target distribution is time dependent such as a Markov chain) or dynamic targets (whose belief distribution may be in more than one cell). We will assume the use of an occupancy grid model [50]. This model has closed form solutions which are obtained by judiciously applying basic properties of probability and information theory; use of other process/sensor models lies beyond the scope of this paper. Define submodular function $I(Z_{k+1:k+q};X|z_{1:k})$ to be the total mutual information gathered by a total of *q* future sensor observations. We note that in this approach, mutual information is used to predict the total information gain of future trajectories conditioned on any previous sensor measurements observed.

Given a particular configuration c∈C, the sensor model also defines a sensor configuration footprint, encoding which cells in E are observed given the perspective (field-of-view), and characteristics of the sensor (consider the projection of the image plane onto the evidence grid for a downward facing camera). This extends to trajectories, defining the trajectory footprint of the sensor following the path of the trajectory (Equation (2)).

(2) $$\Phi_{T}=\left\{\cup_{x,y}Z_{k+1:k+q}^{x,y}|T\;\mathrm{makes}\;q\;\mathrm{observations}\;\mathrm{at}\;\mathrm{cell}\;g_{x,y}\right\}$$

It holds that different sensors have different footprints (e.g., downward facing camera vs. scanning LIDAR). $\Phi_{T}$ is equivalent to a 2D array the same size as the evidence grid by defining $\Phi_{T}\left[x,y\right]$ to be the number of observations in the indexed cell. The cumulative mutual information collected by time *t* assuming the robot has followed $T_{t}$ is given by the sum of the mutual information of the future sensor measurements and the hidden state conditioned on previously observed data within each cell.

$$R\left(T_{t}\right)=\sum_{Z_{k+1:k+q}^{x,y}\in \Phi_{T}}I\left(Z_{k+1:k+q}^{x,y};X^{x,y}|z_{1:k}^{x,y}\right)$$

The problem of multipass coverage planning for information gathering with mission duration constraints is defined as follows: Find $T_{t}^{*}$, the trajectory of time duration *t* that maximizes the cumulative mutual information in $E_{t}$,

(3) $$T_{t}^{*}=\underset{T_{t}\in T_{t}}{\arg\;\max}R\left(T_{t}\right).$$

### 3.1. Motion and Sensor Model

**Figure 2:**
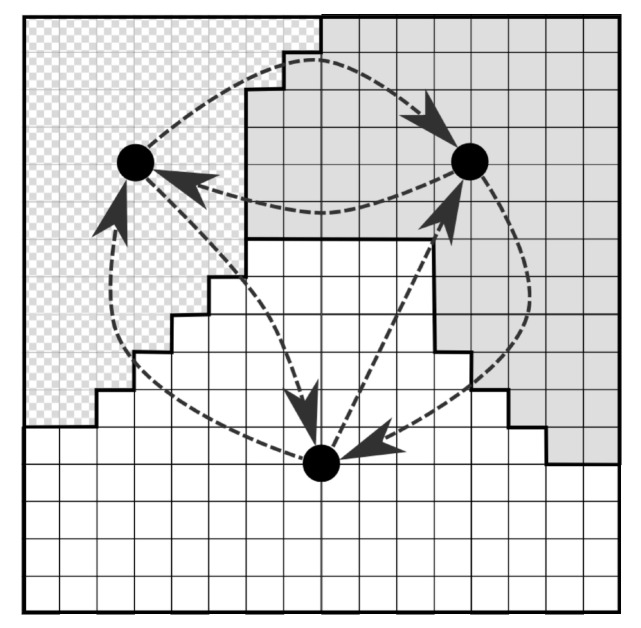
Example grid divided up into 3 connected regions or nodes. The agent’s motion model consists of different regions (also referred to as nodes). Each region contains a subset of cells (white, grey or checkered). A cell may belong to multiple regions, not shown. Each region has a central point (black circle) contained within the cell. The actions available to the agent at a given region is to either search the current region, or move to an adjacent region along a path connecting central points.

We assume a motion model $T_{t_{l+1}}=\psi \left(T_{t_{l}},a_{l}\right)$ where the current trajectory and next action determine the subsequent trajectory (along with next state *c* and trajectory footprint). In order to minimize the number of actions required to generate a coverage plan to minimize the depth at which one must search to find the solution, we cluster the cells of the evidence grid into contiguous, connected regions. The agent then has the choice to either traverse between connected regions, or search a given region. See Figure 2 for an example motion model. At a lower level hidden from the planner trying to solve (Equation (3)), the agent stitches motion primitives together to generate a trajectory to complete a given action (Section 5.3). Previously, we tested the proposed approach in rectangular environments [13]. In this work we extend such findings to more natural simulated Boustrophedon environments as will be covered in Section 6.1 and Section 6.2.

## 4. Preliminaries

Now that we are given a proper problem formulation, we now introduce relevant background material before proceeding to the approach.

### 4.1. Benchmark Algorithms

In addition to our proposed algorithm, there are three additional algorithms we would like to benchmark for the described scenario, that we can classify as being either heuristic solvers or planning algorithms. It is reasonable to suppose that heuristic solvers will get solutions faster by solving an easier problem (often in polynomial time), but will get lower quality solutions. Planning algorithms, on the other hand, solve the problem at hand by reasoning over many candidate solutions. Planning algorithm performance can be improved by using heuristics to guide the search. In practice, the use of properly designed heuristics to guide the search reduces the computational effort by a significant constant factor making exponentially complex algorithms tractable, while still obtaining (near-)optimal solutions.

We evaluate two heuristic solvers including (1) a single step greedy heuristic solver; and (2) the depth first search coverage based planner which is used in covering Boustrophedon decomposed environments [48] and has been adapted to generate multipass coverage plans. For planning algorithms, we test both depth first branch and bound and ϵ-admissible branch and bound (ϵ-admissible B&B: Algorithm 1 [13]). ϵ-admissible B&B uses improved heuristics to dramatically increase the quality of the solutions. Similar to ARA* [51] and *e*-admissible search [52], ϵ-admissible B&B uses an inadmissible heuristic to speed up the search time for branch and bound to find a good solution with bounds on sub-optimality.

The greedy heuristic algorithm greedily maximizes total information gathered per unit of time for the next immediate action. When the greedy heuristic is selecting the next best action, it is possible (but unlikely in natural environments) that two or more actions result in the same (maximal) amount of information gathered per unit of time. Such ties are resolved by selecting the action that acquires more total information. This is done iteratively until the planned trajectory spans the entire mission duration.

For the depth first coverage solver, one uses a modified version of depth first search (DFS) to generate a tree that spans all regions in the environment. Further, the iterative greedy heuristic *g* computes how many times region *i* should be searched $S_{g,i}$ to maximize mutual information. The agent then traverses the tree based on the DFS order, keeping track of how many times it has searched each region $S_{i}$. To ensure a more uniform coverage of the environment, the agent searches the current region at most once per visit. Traversing the DFS tree will have the agent visit most nodes multiple times, and the tree can be traversed multiple times until the time expires (provided there is enough time to search). Each region is searched at most $S_{i}\leq S_{g,i}$.

To illustrate the necessity of using priority heuristics and ϵ-admissible heuristics, we contrast ϵ-admissible B&B to depth first branch and bound (DF-B&B), as this is the more typical implementation of branch and bound (especially when implemented using recursion instead of a queue). Common practices with branch and bound tends to favor a depth first ordering when enumerating partial solutions because it tends to find initial (complete) solutions quicker enabling one to start bounding the space earlier in the search. Best first search strategies, such as A* with good heuristics, will evaluate fewer nodes when looking for the optimal solution however. Note that DF-B&B differs from DFS in that DF-B&B computes $\tilde{g}$ for every node, where DFS finds a spanning tree of all regions which in turn defines the search order, and only computes $\tilde{g}$ once to determine how many times each region should be searched. DFS therefore is much faster to compute than DF-B&B.

Note that by convention our motion model returns actions that traverse regions (sorted based on edge information) before actions that exhaustively search regions. Without modification, DF-B&B will only focus on actions that traverse between many regions and find low quality solutions within the allotted time. To generate high quality solutions quickly, the action set ordering should be randomized (i.e., permuted) such that DF-B&B will deliberate over more diverse action sequences. Such permutation schemes should be complicated enough to prevent cycles in the action space. This modification remains within the scope of the depth first search algorithms since the motion models do not require a preference over the ordering of different actions.

In previous work [13] we used Zelinsky’s algorithm [47] as a benchmark, however Zelinsky’s algorithm does not readily extend to sensor models whose footprints extend beyond a single cell in the grid and hence the sensor models used in this study.

### 4.2. Heuristics

Several challenges exist in designing heuristics for information gathering for multipass trajectories that do not exist in path planning for waypoint following:

- All regions must be explored (possibly multiple times), requiring the heuristic to be more computationally demanding and explore a larger portion of, if not the entire workspace.
- The reward function is *path dependent* since mutual information is submodular, requiring planning in the space of trajectories instead of the configuration space.
- There is an increased number of paths of equal or similar reward, further compounding the problem.

To counter these challenges we define the notion of an ϵ-admissible heuristic for branch and bound in previous work, and observe that at significant performance speedup we can more greedily prune the solution space with a bounded loss in optimality [13]. For our application we effectively transform the admissible iterative greedy heuristic *g* into an ϵ-admissible $\tilde{g}$ (see Section 6).

#### Iterative Greedy Heuristic

For completeness we now describe the iterative greedy heuristic developed for multipass search in previous work [13]. Relax the problem formulation in (Equation (3)) by permitting sequences of actions that are not feasible. In other words, regardless of the agent’s current state, the agent must select *L* discrete actions to take, from all $N_{a}$ actions that are available from any state (Note that to guarantee admissibility $N_{a}$ is the sum of all actions that transition between regions and all actions that search a given region, Section 3.1).

Define decision variable $a_{i}\in \left\{0,1,\ldots ,L\right\}$ with $i\in 1,\ldots ,N_{a}$ the number of times the *i*th action is taken, with decision vector $a=(a_{1},a_{2},\ldots a_{N})$. For the multipass coverage planning problem, actions can be selected multiple times. Selecting action *i* the $\left(a_{i}+1\right)$th time yields the incremental reward of $\Delta R_{i}\left(a_{i}+1\right)$. $R_{i}$ is monotone increasing and concave ($R_{i}\left(a+1\right)\geq R_{i}\left(a\right)$ but $\Delta R_{i}\left(a+2\right)\leq \Delta R_{i}\left(a+1\right)$). The agent’s goal is to select a to maximize the function:

(4) $$\;\;\max_{a}\sum_{i=1}^{N}R_{i}\left(a_{i}\right)\;\;\;\;s.t.\;\;\;\;\sum_{i=1}^{N}w_{i}a_{i}\leq L\;\mathrm{and}\;a_{i}\geq \alpha_{i}\geq 0$$

**Figure 3:**
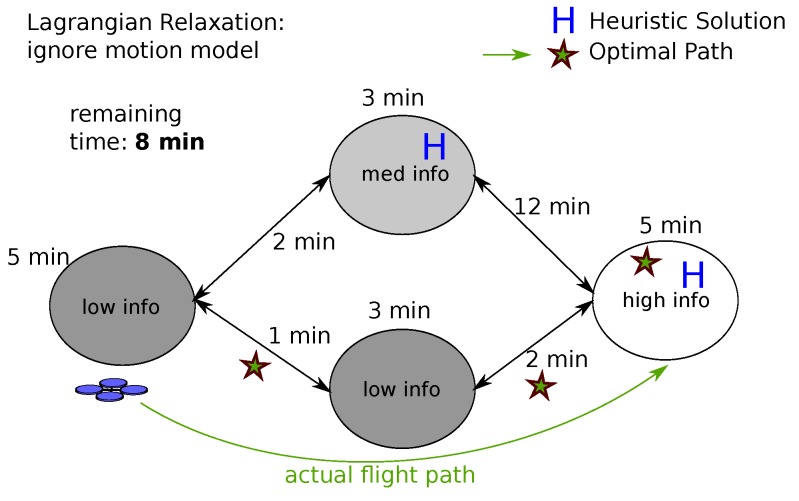
An illustration highlighting how the heuristic is an upper bound. Relaxing the motion model is akin to allowing the agent to move freely between regions ignoring the motion model. The heuristic is able to sample the regions with the most information given the mission duration but ignores path constraints, such as having to navigate the 12 min edge. The optimal solution is to traverse the bottom of the environment.

The strategy to find the optimal solution is to sort all actions based on the incremental reward. While picking one action may affect the reward of other actions, those actions affected are a small subset of all $N_{a}$ actions and can be recomputed, since information content is localized to a given cell, and the information in cells may be accessed by different actions. During each round the action with the most reward is selected; by the concavity of the reward function the reward for taking an action *a* the *i*th time is collected before taking action *a* the (i+1)th time [13]. An illustration of how the iterative greedy heuristic works in practice can be found in Figure 3.

This formulation is similar to previous work that uses multi-armed bandit (MAB) relaxations for gathering information [53]. However, the MAB heuristic computes dynamic allocation indices [54] in scenarios where reward is uncertain. The iterative greedy heuristic gathers expected information gain, which automatically factors measurement uncertainty, offering a simpler approach for the problem at hand.

One of the main limitations to the iterative greedy heuristic is that it does not account for the flight time associated with the agent transitioning between regions. This means that many different distributions of information will have the same heuristic reward even though it will be easier to gather the reward in some scenarios and not in others. Algorithms like A* or best first branch and bound expand all nodes with optimistic reward greater than or equal to the optimal reward, dramatically increasing search time if there are many paths of similar cost. One might consider getting a tighter bound on total information a node can gather with the remaining time available before the mission ends by estimating the total remaining traversal time. One such way to obtain a lower bound is to generate a minimum spanning tree [55] of all regions the heuristic visits. While this approach works for traveling salesman problems [43], such an approach cannot properly bound the information gathered when traversing between regions, making it unsuitable for the problem at hand.

Further, in large maze-like environments, it is often difficult for the planner to explore the search frontier when a candidate solution backs itself into a corner and requires multiple actions to return to the frontier, which is likely to happen when a multipass algorithm is used in large environments.

The iterative greedy heuristic requires the evaluations of many footprints for their information content. In order to speed this up, we first note that changes in cost can be computed locally for the region being evaluated immediately. Second, caching the rewards for footprints is essential to improving the speed of the heuristic. Whenever a region is selected greedily, at the next iteration the rewards only have to be recomputed for the previously selected region (and any other regions it may overlap), resulting in a constant factor performance improvement with $N_{r}$. Caching is therefore a constant factor improvement in the run time of the heuristic.

## 5. Approach

In order to solve (Equation (3)), we benchmarked four different algorithms using the same reward and motion model to determine their ability to generate quality solutions and determine how long it takes them to compute the solution. As we did in previous work [13], we observe that $I(Z_{k+1:k+q};X|z_{1:k})$ can be computed from $I(Z_{k+1};X|z_{1:k})$ as a 3D look-up table dependent on the number of positive and negative observations for a given cell by applying the chain rule for mutual information [4] and the exchangeability property of Binomial random variables, offering O(1) mutual information look-up speeds in RAM with as little as 32 kilobytes of memory for a 20 × 20 × 10 array of double precision floats.

Our proposed method is ϵ-admissible branch and bound [13], is based off of branch and bound [56] and is summarized in Algorithm 1 with subroutines Branch (Algorithm 2) and Bound (Algorithm 3). In short, branch and bound works by iteratively partitioning the solution space (branch), and determining if a subspace contains a solution that can potentially beat the current best known solution using an admissible heuristic (bound). Subspaces that can be determined to not contain the best solution are pruned or *fathomed*, speeding the search. When expanding the search tree denoting subsets of the solution space, define a search node *N* to be a partial solution with a family of candidate solutions, combined with a reference to its parent node, its priority, and other quantities required for improving the search. Search node *N* is different from a node in the graph of the motion model. DF B&B can be created by modifying Algorithm 1 such that $Q_{open}$ is a stack (LIFO queue), priority heuristic P(·,·) is no longer required, line 14 in Algorithm 1 is eliminated, and admissible heuristic *g* is used in place of ϵ-admissible heuristic $\tilde{g}$.

### 5.1. Priority Heuristic

For the priority heuristic in ϵ-admissible branch and bound (Algorithm 1 line 14), we use the weighted heuristic that discounts future reward to offer a compromise between best first and depth first ordering. For a node *N* in the search tree, define priority P(N,g) to be (Equation (5)) [13]:

(5) P(N,g)=R(N)+α(g(N)−R(N))

given current reward R(N), admissible bounding heuristic g(N), and future reward discount factor α∈[0,1]. Setting α towards zero biases the search towards depth-first like behavior.

**Algorithm 1**
ϵ-Admissible Branch and Bound
**Require:** Priority Queue $Q_{open}$, Queue $Q_{closed}$, evaluation function f(·), ϵ-admissible heuristic $\tilde{g}\left(\cdot \right)$, priority heuristic P(·,·), Start node $N_{start}$, end time Tmax, maximum number of iterations Imax, heuristic reward $B_{0}$1:$B\leftarrow B_{0}$2:$Q_{open}.insert\left(N_{start}\right)$3:i←04:**while** $Q_{open}$ is not empty or i<Imax **do**5: i←i+16: $N\leftarrow Q_{open}.pop\left(\right)$7: $Q_{closed}.insert\left(N\right)$8: **if** *N* is a complete candidate solution and f(N)>B **then**9:  B←f(N)                                                //new best solution found. Store it.10: **end if**11: N←Branch(N)12: **for all** $N_{i}\in N$ **do**13:  **if** $Bound(N,B,\tilde{g})$ and (N not in $Q_{open}$ and *N* not in $Q_{closed}$) **then**14:   $N_{i}.priority\leftarrow P\left(N_{i},\tilde{g}\right)$15:   $N_{i}.parent\leftarrow N$16:   $N_{i}.R\leftarrow f\left(N_{i}\right)$17:   $Q_{open}.insert\left(N_{i}\right)$18:  **end if**19: **end for**20:**end while**


**Algorithm 2** Branch(N)
**Require:** motion model ψ1:**for all** available actions *i* from ψ **do**2: T=ψ(N.T,i)      //roll out trajectory using motion model3: $N_{i}.T\leftarrow T$            //append to new search node4:**end for**5:**return** $\left[N_{1},N_{2},\ldots ,N_{m}\right]$


**Algorithm 3** Bound(N,B,g)
**Require:** Search node/partial solution *N*, current best reward *B*, heuristic g(·)**Ensure:** determination if *N* should be expanded.1:**if** g(N)>B **then**2: **return**       True //current partial solution can potentially beat best known solution3:**else**4: **return**          False //current partial solution cannot beat best known solution5:**end if**


### 5.2. Agent Model

While this work is targeted at quadrotors, the outlined approach generalizes to other physical systems. Although quadrotors are a differentially flat system [57] requiring 4th order dynamics, we will focus on using a second order integrator subject to velocity and acceleration constraints for this work. We first observe that physical systems have bounds on their acceleration capabilities due to the force output of their actuators, or the maneuvers their controllers can reliably execute, imposing a maximum nominal acceleration on the platform $a_{max}$. Lower accelerations for quadrotors reduces the maximum attitude angles observed (similarly with their derivatives), which can affect sensor measurements. Second, we observe that it is often the stopping distance vs. the range of onboard collision detection sensors that dictates how fast a vehicle can safely fly in partially known environments, requiring a hard bound on maximum nominal velocity $\upsilon_{max}$. When traveling at maximum speed $\upsilon_{max}$, decelerating at $a_{max}$, the stopping distance before the system comes to rest is $d_{stop}=\frac{\upsilon_{max}^{2}}{2a_{max}}$. The vehicle can safely stop if its stopping distance is shorter than its sensing range. Alternatively, if the vehicle wishes to steer around an obstacle $d_{obs}$ distance ahead maintaining forward speed, the distance the robot can steer, $d_{steer}=\frac{a_{max}d_{obs}^{2}}{\upsilon_{max}^{2}}$. The robot steers clear if $d_{steer}$ exceeds the distance it must move to avoid collision. In short, a vehicle that wishes to double its safe forward speed must quadruple its acceleration capabilities or sensing range. Further, analysis of single order integrator systems flying through Poisson forests suggest that above a certain speed, collision will occur with probability one regardless of the planner or controller used [58].

Define ||·|| as the L2 norm while $r={\left[r_{x},r_{y}\right]}^{\prime }$. Other flat states such as altitude or yaw could be included but are ignored for our work.

(6) $$\begin{array}{cc} \ddot{r}= & u\;s.t.\\ & ||\dot{r}\left|\right|\leq \upsilon_{max}\\ & \left||u|\right|\leq a_{max} \end{array}$$

### 5.3. Motion Primitives

In order to execute a high level behavior, sequences of waypoints $r_{i}$ are chained together to create multiple point to point maneuvers. For now we assume that a decomposition algorithm divides the environment into contiguous regions that are easy to search (how we do this is covered in Section 6.2). When traversing between regions (central point to central point), a simple line connecting the regions may not suffice due to the presence of obstacles. To solve this, we create an alternate motion model that is an 8 connected graph on the underlying evidence grid. A* can generate a sequence of cells minimizing path cost (path length). However, this sequence of waypoints is very dense; many of the waypoints are unnecessary to define the shape of the path and can be eliminated by the following method. One only keeps a waypoint $r_{i}$ in the sequence if the angle between vectors $\left(r_{i}-r_{i-1}\right)$ and $\left(r_{i+1}-r_{i}\right)$ is above some threshold, e.g., $5^{\circ }$. Otherwise, remove waypoint *i* and continue.

**Figure 4:**
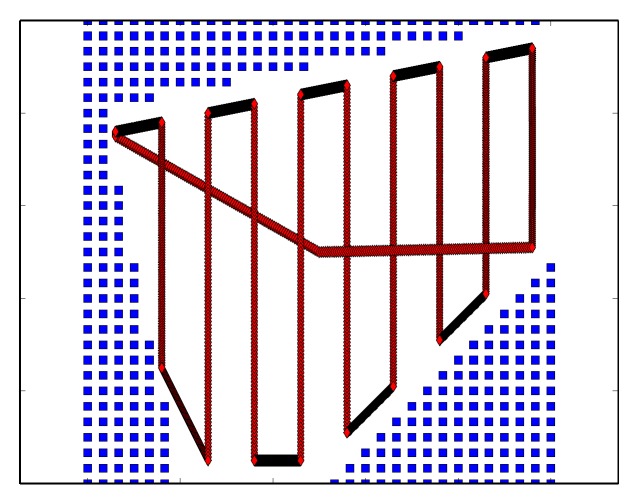
Example generation of rectilinear coverage plan for a region with continuous velocities.

In order to search a region, a dynamically feasible coverage plan is needed. The proposed method creates rectilinear uniform coverage plans (Figure 4) with “plow lines” that are parallel to the slice direction defined by the Boustrophedon Decomposition algorithm (Section 6.2). The agent transitions between adjacent “plow lines” rectilinearly. In order to compute the start and stop positions of the plow line, the geometry of the (boustrophedon) region is known, and line search is conducted to find the max and min values along a given plow line. Spacing between plow lines is dictated by the maximum sensing radius.

The use of a uniform coverage plan makes the tacit assumption that information is uniformly distributed within the region. This may or may not be the case in practice, reducing the effectiveness of the information gathering algorithm. Having a coverage planner that can directly reason over contiguous nonuniform regions is compatible with the proposed approach, but is to be considered in future work. An alternate approach is to use the distribution of information to guide the selection of regions. The mere act of intelligently decomposing the environment into separate regions can dramatically simplify the problem by solving information gathering problems in smaller contiguous regions. We note that for illustrative purposes, all algorithms benchmarked in this paper have access to the same motion model, suffering similarly.

Apart from rectilinear coverage plans, one might consider using spirals [37] due to their ability to efficiently cover 2D areas. However, given the problem specification of having to account for obstacles while generating dynamically feasible coverage plans, The task of covering non-circular/non-square regions found from a decomposition algorithm makes spiral based coverage plans impractical due to inefficiencies in covering the edges.

### 5.4. Point to Point Maneuver

For vehicle trajectory r(t) connecting points $r_{0}$ and $r_{1}$ for times $t_{1}>t_{0}$, solve the following optimization problem:

(7) $$\begin{array}{c} \min_{u}\;t_{1}-t_{0}\;\mathrm{subject}\;\mathrm{to}\;(6)\;\mathrm{and}\text{:}\\ r\left(t_{0}\right)=r_{0}\\ r\left(t_{1}\right)=r_{1}\\ \dot{r}\left(t_{0}\right)=\ddot{r}\left(t_{0}\right)=\dot{r}\left(t_{1}\right)=\ddot{r}\left(t_{1}\right)=0 \end{array}$$

For the system in (6), the problem in (Equation (7)) is a simple bang-bang control problem [59] minimizing the trajectory time interval $t_{1}-t_{0}$ by selecting optimal control u. The corresponding trajectory can be written in closed form with continuous position and velocity, and piece-wise constant acceleration at $a_{max}$. The system either has one or two switching times depending on whether the system has enough time to accelerate to its maximum velocity.

Without loss of generality we will consider a scalar model, letting $r=r_{x}$ with dynamics $\ddot{r}=u$. Let $\Delta r=r\left(t_{1}\right)-r\left(t_{0}\right)$, $\dot{r}\left(t\right)=\upsilon \left(t\right)$ and $\ddot{r}\left(t\right)=a\left(t\right)$.

**Theorem** **1.** Necessary condition: the time optimal control resides within the domain $u\in \{-a_{max},0,a_{max}\}$.

**Proof.**  There are two cases, when there exists 1 switching time (when $|\upsilon \left(t\right)|<\upsilon_{max}$ and t is not the switching time or when $\left|\Delta r\right|\leq r_{crit}$) or when there are 2 switching times ($|\Delta r|>r_{crit}$) for $r_{crit}=\frac{\upsilon_{max}^{2}}{a_{max}}$. When $\left|\Delta r\right|\leq r_{crit}$, one can show by using the Pontryagin’s Maximum Principle (PMP) [59] that control for the time optimal solution $u\in \{\pm a_{max}\}$.

When $|\Delta r|>r_{crit}$, the velocity constraint will become active during the trajectory. As a simple illustration, suppose that the system accelerates to the maximum velocity $\upsilon_{max}$. To continue accelerating would violate the velocity constraint, so the system must either stop accelerating or slow down. For any system slowing down before the prescribed deceleration at the end of the trajectory, the traversal time would increase, yielding a suboptimal solution to the time optimal problem. Therefore, the system will cruise at $\upsilon_{max}$ with u=0 until it is time to slow down and $u\in \{-a_{max},0,a_{max}\}$. ☐

For a more rigorous proof, consider a formulation of Pontryagin’s Maximum Principle with state inequality constraints to get the case when the velocity constraint becomes active. Typical formulations of PMP without inequality constraints will not elicit costate trajectories that have multiple switching times.

With the domain of *u* and known problem constraints, the switching times can be determined along with the optimal control by inspection (speed up, cruise, slow down). With known control *u*, υ(t) and r(t) can be solved for closed-form by quadrature. To increase the average speed of the vehicle, it is advantageous to remove the zero velocity and acceleration constraints at the end of each maneuver. Boundary conditions with $\ddot{r}\left(t_{0}\right),\ddot{r}\left(t_{1}\right)\neq 0$ are easy to solve for closed form using the mentioned approach when the velocity is in the direction of the vector $r\left(t_{1}\right)-r\left(t_{0}\right)$. Two extensions are to use splines (where it is difficult to guarantee hard bounds on velocity and acceleration) or to use Dubbins like trajectories where the use of circles connect adjacent plow lines.

### 5.5. Search Effort Allocation Model

For comparison of results between algorithms, we define a simplified search effort allocation model to see how effective the various multipass coverage planning algorithms are at directing rescue teams. This simplified model can study the practical consequences of having informative trajectories for search and rescue but ignores effects due to finite search resources/time, survivor survival rates, and the time spent transitioning between cells. The search effort allocation model is *distinct* from the sensor model used by the planner based on the different attributes or roles the autonomous agent and human ground crews have during the search and rescue operation. We assume that after executing the autonomous search using one of the benchmark algorithms outlined in Section 4.1, the agents generate an occupancy map which become prior probabilities $P_{0}=\mathrm{Pr}\left(X=1\right)$ for the human rescue teams on the ground. Let *D* be the event that the target is detected within a given cell, while ¬D is the absence of detection. *X* is the presence or absence of a target in the cell as in the previous model (Equation (1)). τ is the detection time constant effectively rescaling the given search time to search effort. Define the probability of detection of a target in a given cell (Equation (8)) [5,60]:

(8) $$\mathrm{Pr}\left(D|X=1,t\right)=1-e^{-t/\tau }$$

Using Bayes’s rule and (Equation (8)), we compute the probability the target is still present despite there not being a detection after searching for *t* seconds (Equation (9)):

(9) $$\mathrm{Pr}\left(X=1|\neg D,t\right)=\frac{P_{0}e^{-t/\tau }}{1-P_{0}+P_{0}e^{-t/\tau }}$$

The human rescue teams search the cell for *T* units of time for a survivor until the survivor is found or $\mathrm{Pr}\left(X=1|\neg D,t\right)<P_{neg}$. If $P_{0}<P_{neg}$, the cell is skipped. Using this policy, the maximum search time $T_{neg}$ for the cell is (Equation (10)):

(10) $$T_{neg}=-\tau \log\frac{P_{neg}\left(1-P_{0}\right)}{P_{0}\left(1-P_{neg}\right)}$$

Note that the probability density function $f_{T|X=1}\left(t\right)=\frac{e^{-t/\tau }}{\tau }$ is due to (Equation (8)). We wish to compute the expected time for the search in a given cell to end by letting $E\left[T\right]=\sum_{p}E\left[T|P_{0}=p\right]\mathrm{Pr}\left(P_{0}=p\right)$ for a given trajectory generated by the algorithm. We start off by computing the expected time to search the cell given prior probability $P_{0}$$E[T|P_{0}]$ by using the law of total expectation over *X* (Equation (11)):

(11) $$\begin{array}{c} E\left[T|P_{0}\right]=E\left[T|P_{0},X=0\right]\mathrm{Pr}\left(X=0\right)+E\left[T|P_{0},X=1\right]\mathrm{Pr}\left(X=1\right)\\ E\left[T|P_{0}\right]=T_{neg}\left(1-P_{0}\right)+\left(\tau -e^{-T_{neg}/\tau }\left(T_{neg}+\tau \right)\right)P_{0} \end{array}$$

This leaves the computation of the distribution of priors $P_{0}$. For each cell in the evidence grid, the distribution of outcomes for $P_{0}=\mathrm{Pr}\left(X=1|K=k,z_{1:k}\right)$ given the sensor data (planned *K* sensor measurements) can be computed for all realizations. $P_{0}$ can be computed directly/deterministically from the known number of sensor measurements and known sensor data $z_{1:k}$. Since we assume that all cells have the same characteristics, we marginalize over the distribution of sensor measurements (computed from the trajectory footprint) and marginalize over all realizations. We also use the exchangability property of the sensor measurements to use a Binomial random variable $M_{k}=\sum_{i=1}^{k}Z_{i}$ (Equation (12)):

(12) $$\mathrm{Pr}\left(P_{0}=p\right)=\sum_{i,k}\mathrm{Pr}\left(X=1|K=k,M_{k}=m_{i}\right)\mathrm{Pr}\left(M_{k}=m_{i}|N=k\right)\mathrm{Pr}\left(K=k\right)$$

Without loss of generality we assume τ=1.0, meaning *t* implies the number of time constants spent searching a given cell.

## 6. Experimental Setup

We now discuss the formulation for modeling quadrotors with downward facing cameras in natural environments, and discuss experimental setup. When selecting our ϵ-admissible heuristic for ϵ-admissible branch and bound we only want the algorithm to expend effort when there may exist a solution that is at least a factor 1 + η better than the current best known solution. We let $\tilde{g}\left(x\right)=g\left(x\right)-\eta B$ where *B* is the reward of the current best known solution. B←−∞ at start up if no heuristic solution is known or computed. Unless otherwise stated we let η=0.5%. Simulations were run on a workstation laptop running 64 bit Ubuntu 14.04 LTS with a Core i7-6920HQ which has a 2.9 GHz clock with 64 GB RAM. The proposed branch and bound algorithm, the greedy algorithm and the depth first search (DFS) coverage planner were developed in Python, using NumPy [61] for array operations and networkx [62] for graph operations.

### 6.1. Procedurally Generated Natural Environments

**Figure 5:**
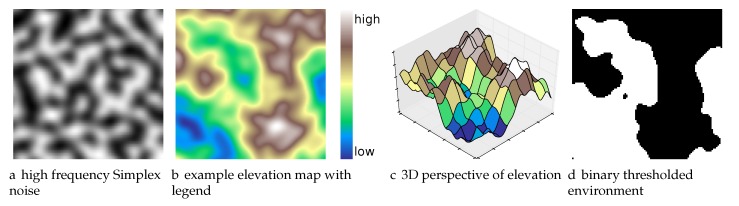
Example Simplex noise using OpenSimplex noise and illustrations on generating natural environments. (**a**) shows 1 frequency band of Simplex noise, while multiple frequency bands create the environment in (**b**–**d**). The color elevation map in (**b**).

In order to procedurally generate natural environments, we use multiple frequency bands of a gradient noise function (for a single band: Figure 5a) to generate elevation maps (Figure 5b is a colored elevation map, while Figure 5c is a 3D perspective of the colored elevation map in Figure 5b), and then threshold that map to create an obstacle grid (Figure 5d). In contrast to Perlin noise, Simplex noise (Figure 5a) has minimal directional artifacts making it most suitable for natural terrain [63,64] (we use the OpenSimplex algorithm developed by Kurt Spencer [65] and ported to Python by A. Svensson [66]).

The frequency content and hence the complexity of the environment can be controlled by summing multiple frequency components (e.g., Figure 5a). OpenSimplex defines the 2D gradient noise function S(x,y,seed) where $\left(x,y\right)\in R^{2}$ but we restrict to integer grid coordinates given a seed value for the random number generator. $T\left(x,y,\mathrm{seed}\right)=\sum_{i=1}^{n_{f}}w_{i}S\left(f_{i}x,f_{i}y,\mathrm{seed}\right)$ defines the elevation map. $n_{f}$ is the number of frequency components in the terrain generator, and $f_{i}$ is the frequency of the *i*th band and $w_{i}$ is the weight of the *i*th band.

For our experiments, we set $n_{f}=2$, $w_{1}=1$ and $w_{2}=0.25$. Define 5 different environment frequency contents: very low frequency (freq.) (vlf), low freq. (low), medium freq. (med), high freq. (high) and very high freq. (vhf). For (vlf,low,med,high,vhf), the frequencies $f_{1}$ correspond to (0.015,0.03,0.045,0.06,0.75) and the frequencies $f_{2}$ correspond to (0.05,0.1,0.15,0.2,0.25). For each environment complexity, our database of benchmark environments consisted of 20 Simplex environments and 4 random starting locations per environment. Algorithm 4 outlines the approach used to generate Simplex environments. This is contrasted to defining random obstacles over, e.g., randomly generated quadtree maps [67].

**Algorithm 4** Generate_Environments(*T*)
**Require:** Noise function *T***Ensure:** set of environments1:Q←∅2:**for all** RNG seed *i* from 1 to *N* **do**3: **for all** coordinates (x,y) in the environment **do**4:  $E_{i}\left[x,y\right]\leftarrow T\left(x,y,i\right)$5: **end for**6: Threshold $E_{i}$ such that 66% of the workspace is collision free.7: Select the largest connected component and set it as free space. Make all other regions obstacles.8: **if** amount of free space is within tolerance **then**9:  Remove obstacles below the minimum size threshold in pixels in $E_{i}$.10:  add $E_{i}$ to *Q*11: **end if**12:**end for**13:**return** *Q*


### 6.2. Boustrophedon Decomposition

Given an environment, the free space must be decomposed into individual components that can be searched or “plowed” using the Boustrophedon (i.e., “the way of the ox”) decomposition algorithm [48]. We use a discrete implementation with the following modifications:

- We ignore small obstacles under a given pixel count. We argue that small obstacles smaller than the sensing radius do not adversely affect the coverage plan yet including a small convex obstacle introduces 3 additional regions to the decomposition.
- We impose a greedy merge rule to combine adjacent regions if their boundaries overlap sufficiently due to critical points far away causing otherwise continuous regions to be broken into smaller regions.
- We also remove regions that are too small to sensibly cover and repair region connections afterwards.
- Since we will be generating trajectories that cover the region, the Boustrophedon algorithm identifies start and end points for each plow line for the trajectory planner.

**Figure 6:**
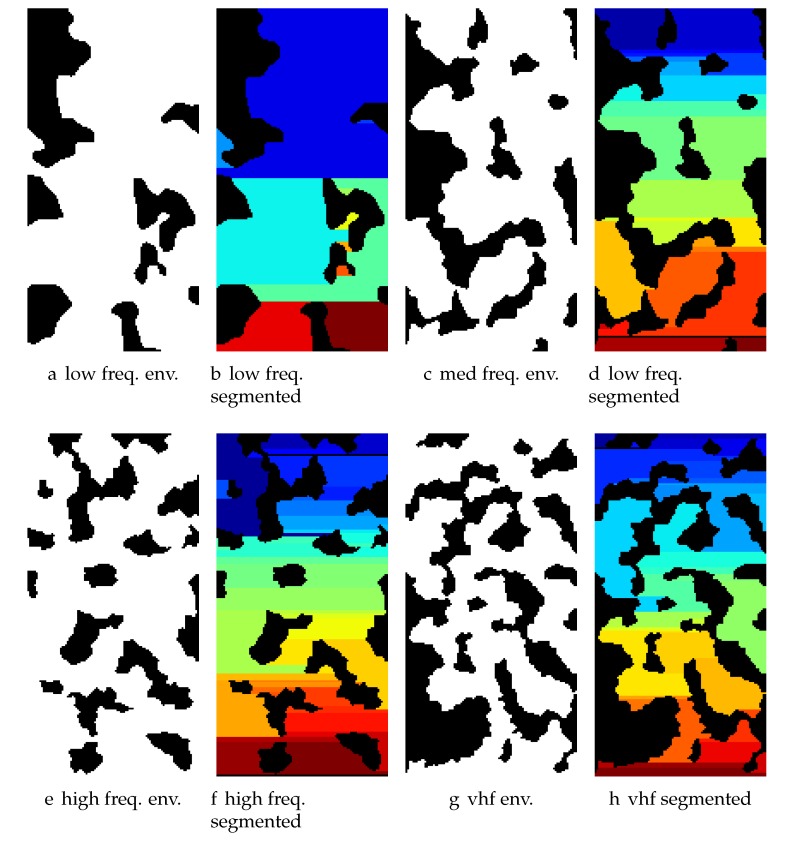
Example environments generated at various frequencies. Black denotes obstacles, and white denotes free space. The white space is divided up into plowable regions by the Boustrophedon algorithm. Each color corresponds to a region.

For the first N=40 computed environments, for the frequency content environments (low,med,high,vhf), the average/standard deviation of number of regions was (13±4.3,28±5.1,37±5.8,45±6.8). Simplex environments are more complex than previous environments [13] due to increased number of loops, and the fact that region traversals gather a significant amount of reward and create significant overlaps between actions. Observe in Figure 6 that the regions vary widely in size given the complexity of the environment. Having fewer nodes in the environment is advantageous in speeding up the search. Larger contiguous regions can be searched more efficiently by acceleration limited agents (Section 5.2). One disadvantage to having such large regions is that the information content of the region may not be uniformly distributed, and the coverage planner may not be able to account for this.

### 6.3. Camera and Sensor Model

For our experiments, we will restrict the sensor model to a binary Bayes static occupancy grid model [50] where P(Z|X) is fully characterized by probability of detection $P\left(Z=1|X=1\right)=p_{d}$ and false positive rate $P\left(Z=1|X=0\right)=p_{f}$. We let $\left(p_{d},p_{f}\right)=\left(0.85,0.15\right)$. Unless otherwise mentioned the target evidence grid E is a 200 × 100 grid.

When computing the recursive Bayes filter (1) for the binary Bayes static occupancy grid model, define $\theta_{k}=P\left(X=1|z_{1:k}\right)$ with $\theta_{0}$ defined using prior knowledge (we typically assume least informative prior $\theta_{0}=0.5$) for new sensor observation $z_{k}$, resulting in (13):

(13) $$\theta_{k}=\frac{P\left(Z_{k}=z_{k}|X=1\right)\theta_{k-1}}{P(Z_{k}=z_{k}|z_{1:k-1})}$$

This results in parameter update equation (Equation (14)):

(14) $$\theta_{k}=\left\{\begin{array}{cc} \frac{\left(1-p_{d}\right)\theta_{k-1}}{\left(1-p_{d}\right)\theta_{k-1}+\left(1-p_{f}\right)\left(1-\theta_{k-1}\right)} & \mathrm{if}z_{k}=0\\ \frac{p_{d}\theta_{k-1}}{p_{d}\theta_{k-1}+p_{f}\left(1-\theta_{k-1}\right)} & \mathrm{if}z_{k}=1 \end{array}\right.$$

In order to compute the multi-step look-up table $I(Z_{k+1:k+q};X|z_{1:k})$ for the binary Bayes static occupancy grid model, we can take a more direct approach using a Binomial random variable to represent the next *q* observations instead of using the chain rule of mutual information. Let $M_{k,q}=\sum_{i=k+1}^{k+q}Z_{i}$ be a Binomial RV where:

Pr(Mk,q=m|X)=qmPr(Z=1|X)mPr(Z=0|X)q−m,form∈0,1,…q

with $\mathrm{Pr}\left(X=1\right)=\theta_{k}$ known from the Bayes filter, $\mathrm{Pr}\left(M_{k,q}=m\right)=\mathrm{Pr}\left(M_{k,q}=m|X=0\right)\mathrm{Pr}\left(X=0\right)$ $+\mathrm{Pr}\left(M_{k,q}=m|X=1\right)\mathrm{Pr}\left(X=1\right)$ using law of total probability. Mutual information for discrete random variables *X*,*Y*$I\left(X,Y\right)=\sum_{x,y}f_{X,Y}\left(x,y\right)\log_{2}\left(\frac{f_{X,Y}\left(x,y\right)}{f_{X}\left(x\right)f_{Y}\left(y\right)}\right)$ with $\mathrm{Pr}\left(X=x,Y=y\right)=f_{X,Y}\left(x,y\right)$ being the joint probability mass function of X,Y and $f_{X}\left(x\right),f_{Y}\left(y\right)$ being the marginal probability mass functions of X,Y. Manipulation yields (15):

(15) $$I\left(Z_{k+1:k+q};X|z_{1:k}\right)=\sum_{m,x}f_{M|X}\left(m|x\right)f_{X}\left(x\right)\log_{2}\left(\frac{f_{M|X}\left(m|x\right)}{f_{M}\left(m\right)}\right)$$

Assuming the initial distribution Pr(X=1)=0.5, $p_{d}=0.85$ and $p_{f}=0.15$, we compute the following table I[nz0,nz1,q] used in the experiments as shown in Table 1.

Observe that when *nz*0 =*nz*1, the mutual information for q=1 is a constant 0.390. This is a limitation of the sensor model itself and is unlikely to occur for large nz0+nz1 when the static binary Bayes sensor model accurately models the environment and $p_{d},p_{f}\neq 0.5$. Since *X* is Bernoulli there is at most 1 bit of information. The table is also symmetric when swapping nz0 and nz1 because $p_{d}=1-p_{f}$. Note that since mutual information is submodular, I[nz0,nz1,2]<2·I[nz0,nz1,1].

**Figure 7:**
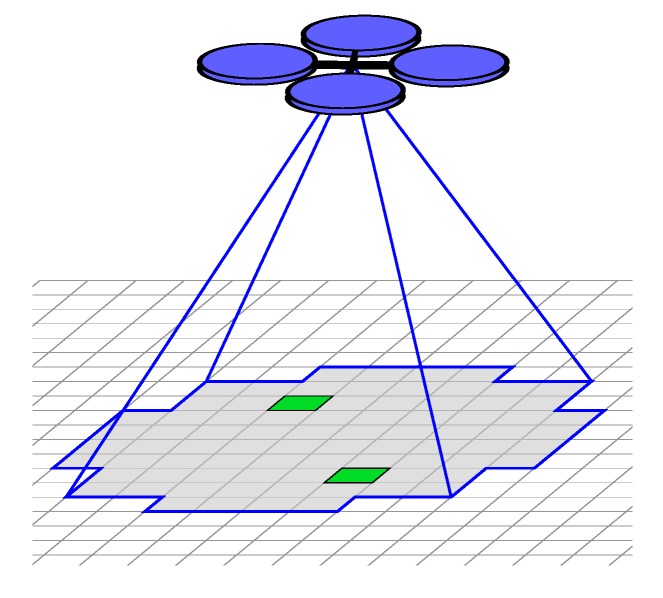
Sensor model for downward facing camera. Green/dark squares indicate cells containing detected targets, while grey squares indicate cells that are within the field of view with no target detections. Targets are not shown.

For this work, we assume that the quadrotor has a downward facing camera with a circular field of view and can make observations in multiple cells (Figure 7). We suppose that there exists image processing software that will identify the positive positions of detected targets in the image frame at a near continuous rate (overlap of FOVs of adjacent images processed is significant), while empty positions of the frame are considered negative detections. We assume that the probability of detection $p_{d}$ and false positive rate $p_{f}$ are known. These locations are then mapped from the image frame to the evidence grid. To avoid multiple correlated sensor measurements we only record a single observation for a given cell during a particular action (transition between regions or search a region). This implies that targets in cells within a given distance from the quadrotor can be detected.

For realistic flight scenarios factoring actual hardware capabilities, motion blur on a global shutter camera occurs when camera/environment motion causes the displacements of image features during the image exposure to be greater than one pixel. Both rotational and translational velocities of the quadrotor contribute to motion blur, placing constraints on both the altitude and forward speed of the vehicle to prevent motion blur. To compare, we will consider the IDS UI-3251LE camera with optics that have a 90deg FOV (7 mm focal length lens), 1600(H) × 1200(V) pixels, flying at 5–20 m altitude, and a 1 ms exposure time (suitable for brightly lit indoor environments) and outdoor environments.

Define $\upsilon_{g,max}$ to be the maximum speed of the ground in the image plane where $\upsilon_{g,max}=|\upsilon_{max}|+r_{z}\left|\omega_{max}\right|$ assuming nominal altitude above the ground $r_{z}$, and $\omega_{max}=0.5\frac{\mathrm{rad}}{s}$ is the maximum attitude angle change expected of the camera during maneuver execution or disturbance rejection. We also note that $FOV=\frac{r_{z}d_{sen}}{d_{foc}}$ where $d_{foc}$ is the lens focal length and $d_{sen}$ is the size of the image sensor along the direction of motion [68].

Define the displacement δp of the ground plane due to agent motion within the exposure time $t_{exp}$ in pixels (Equation (16)):

(16) $$\delta p=\upsilon_{g,max}t_{exp}\frac{N_{p}}{FOV}$$

with $N_{p}$ as the number of pixels along the direction of motion and FOV is the field of view in meters along the direction of motion. δp≥1 indicates motion blur. We note that an increase in altitude $r_{z}$ reduces motion blur by increasing pixel size on the ground but rotational disturbances will dominate for high altitudes. A gimballed camera can reduce rotational disturbances at increased payload costs. This results in the permissible flight envelope by $\upsilon_{max}\in \left[0,10\right],r_{z}\in \left[5,20\right],\delta p<1$. For example, setting $\left(\upsilon_{max},r_{z},t_{exp}\right)=\left(10,10,2\times 10^{-3}\right)$ results in a δp=1.8 causing motion blur while $\left(\upsilon_{max},r_{z},t_{exp}\right)=\left(20,5,2\times 10^{-3}\right)$ results in δp=0.9 not causing motion blur.

We define cell pitch r=2.2 m (not to be confused with camera pixel pitch) mapping cell indices to Euclidean coordinates in meters. We also assume that the camera has a circular 90deg FOV such that the sensing radius is 8.8 m. The trajectory footprint of every action in the motion model is created by sweeping the sensor footprint along the trajectory planned by the Boustrophedon coverage planner. Therefore in practice the shape of the field of view does not matter as the sensor acts as a sweep sensor with the given sweep radius.

## 7. Experimental Results

For each algorithm we normalize its performance with respect to the iterative greedy heuristic which always overestimates the reward. In other words, one computes the ratio of information each algorithm gathers in an environment by dividing the amount of mutual information (in bits) the algorithm gathers by the heuristic reward (in bits). Since the heuristic is admissible this ratio is ≤100%. This also normalizes for the variations in how much total information exists in a given environment. We ran 3 experiments; the first two were to understand various aspects about ϵ-admissible B&B.

- Experiment 1: vary α∈{0.2,0.7,0.8,0.9}, and η∈{0%,0.5%,1.0%,2.0%,3.0%} in low frequency environments for ϵ-admissible B&B to identify algorithm performance for different heuristic parameters.
- Experiment 2: setting (α,η)=(0.8,0.5), vary size of evidence grid in low frequency environments and observe how ϵ-admissible B&B scales with the evidence grid size.
- Experiment 3: benchmark the four algorithms in Simplex environments, setting (α,η)=(0.8,0.5) for ϵ-admissible B&B.

For uniform environments, all cells are initialized with the most uninformative prior of $P\left(X=1\right)=\theta_{0}=0.5$. By inspecting the mutual information look up table, we observe that cells that have two or more prior sensor measurements have low enough information content that a nonuniform covering will gather more information than a uniform covering. When generating nonuniform environments, we want the geometry of low entropy regions to not correspond with the geometry of the obstacles. Therefore the nonuniform envionments generate a distribution of low entropy regions using Simplex noise thresholded at 50% (i.e., 50% of the environment is low entropy, while the remaining environment is set to high entropy).

For the environments (low,med,high,vhf), define the low entropy regions with the following frequency parameters (vlf,vlf,low,med).

### 7.1. Experiment 1

**Figure 8:**
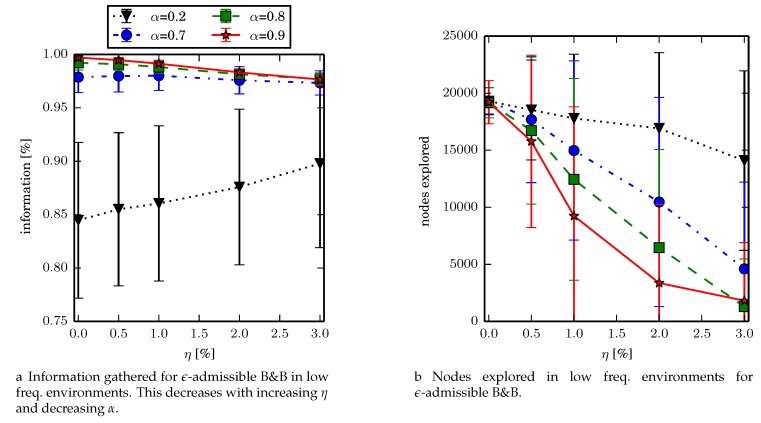
Results for Experiment 1.

We tested the branch and bound algorithm in the low environment, terminating if the priority queue is empty or after 6000 iterations have occurred. We set α∈{0.2,0.7,0.8,0.9}, and η∈{0%,0.5%,1.0%,2.0%,3.0%} and observe total information gathered, and number of nodes explored. Fewer nodes explored implies more nodes in the search space are pruned. As both α and η increase, the number of nodes explored decreases due to better prioritization or pruning. Results are summarized in Figure 8. In terms of the reduction in nodes expanded, for (α,η)=(0.9,1.0%) on average only expands 48% of the nodes that are expanded when (α,η)=(0.9,0.0%) suggesting that ϵ-admissible branch and bound can speed up the search by about a factor of two while finding solutions that are on average within 0.6% of the optimal solution.

In determining the parameters for future experiments, (α,η)=(0.8,0.5) was chosen for the following reasons . Setting η=0.5% ensures that ϵ-admissible B&B finds solutions within 0.2% of the optimal solution. While α=0.9 expands the fewest nodes in the lowf environments, in larger environments, setting α=0.8 instead of α=0.9 decreases the time to finding the first solution which significantly increases the success rate of the algorithm and reduces memory usage (Experiment 3).

### 7.2. Experiment 2

For Experiment 2 we varied the size of the evidence grid to consist of $N_{cells}\in$ {4608, 10,368, 20,000, 39,200, 80,000, 180,000, 320,000 } where each environment has a 2:1 aspect ratio. In addition, the resolution of the cells changes such that the area of the environment in meters is the same in all trials. Also, the sensor footprint is the same in meters in all environments. For the Experiment 2 Simplex environments, the following parameters are: $\left(f_{1},f_{2}\right)=\left(0.0075,0.1\right)$ and $\left(w_{1},w_{2}\right)=\left(1,0.0625\right)$.

For each environment size we generated 10 environments with 4 starting locations for a total of 40 trials. Environments are generated using Simplex noise for environments with 320,000 cells (or 800 × 400 cells) and are downsampled to smaller environment sizes.

**Figure 9:**
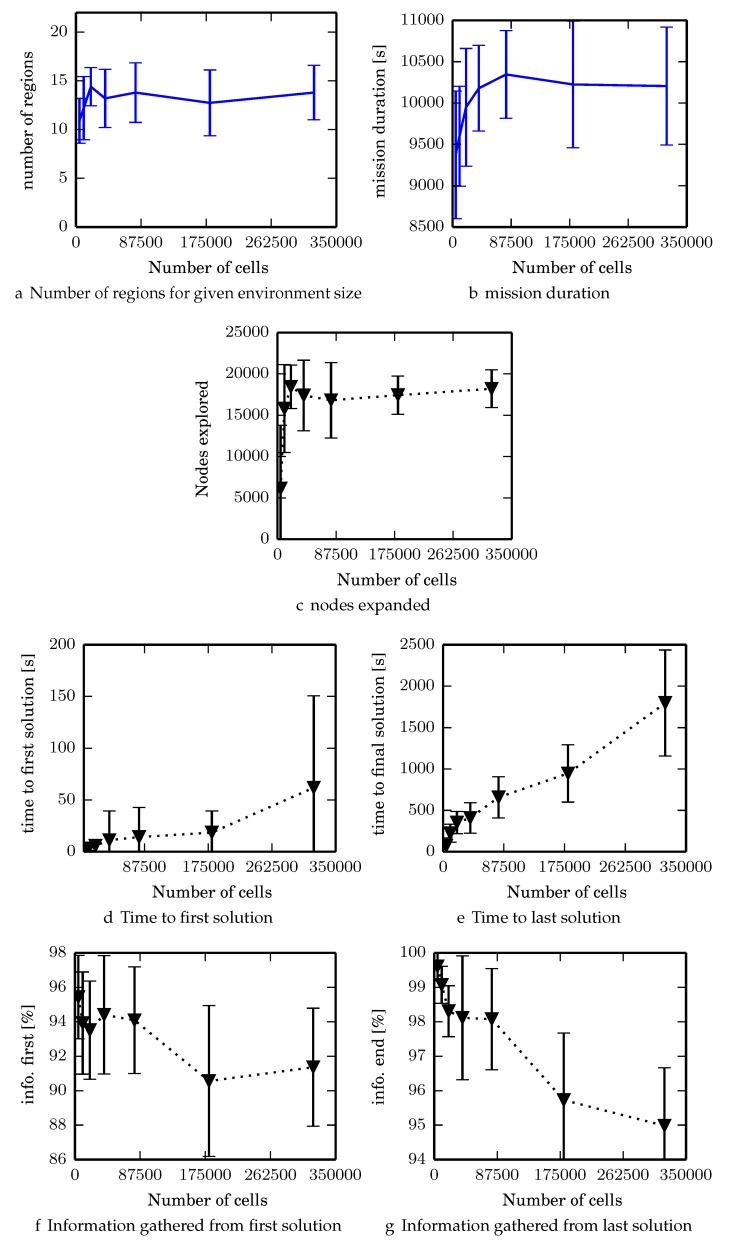
Results for Experiment 2.

After the environment is re-scaled, Boustrophedon decomposition generates the discrete graph before running the proposed branch and bound algorithm. The algorithm also scales parameters for Boustrophedon decomposition such as the minimum area of an obstacle before it is removed. The environments do not scale perfectly, however, and the number of regions per environment can still vary (Figure 9a). Note that the size of the environment in cell count varies by a factor of 70, while the number of regions varies by a factor of 1.3, implying that the increase is insensitive to increase in cell count. Also note that $T_{max}$, defined by the total time to cover each region twice, is insensitive to the number of cells and its standard deviation over all cell sizes is about 750 s with a mean of 9980, varying by about 7.5% (Figure 9b). Branch and bound runs for 6000 iterations or until $Q_{open}$ is empty. The time to the final solution is approximately linear (Figure 9e) when the number of nodes expanded remains constant (Figure 9c).

**Figure 10:**
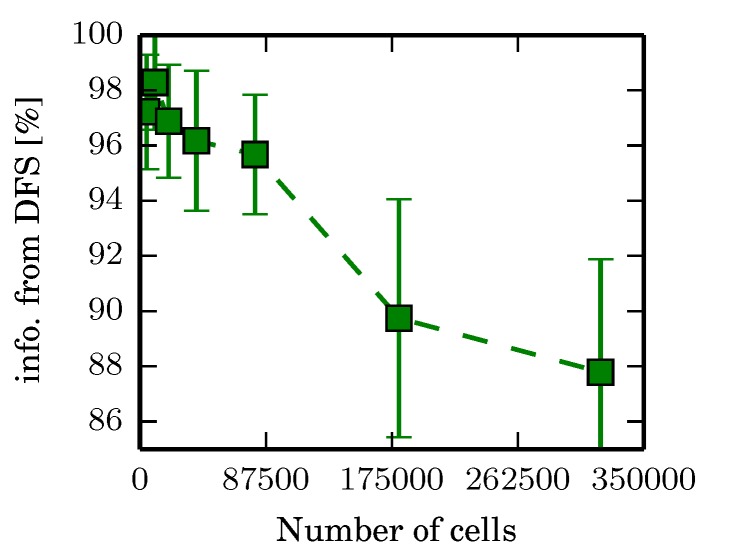
Information gathered from DFS. This is a benchmark to demonstrate that solution quality degrades with $N_{cells}$ and is not dependent on the algorithm.

One thing of interest is that the quality of the solution degrades as the size of the environment increases (Figure 9f,g). This is not a limitation of the algorithm itself; Figure 10 shows that the quality of solutions found using DFS on the same environments degrades similarly with evidence grid size. One possibility that explains this is that the environment is not scaling perfectly. When the sensor footprint is discretized in a coarser grid (when $N_{cells}$ is smaller resulting in larger grid resolutions), the footprint tends to be more square. This can introduce artifacts in the total area swept by a moving sensor by a factor at most $\sqrt{2}$ when moving diagonally, dramatically increasing how much information per unit of time is gathered when traversing between regions. Traversals in environments with fewer cells would therefore get a slight performance boost.

Note that in certain environments, many of the resulting statistics are heavily skewed and non-Gaussian so the error bars (Figure 9d) which represent one standard deviation dip below 0. None of the trials violate constraints such as t<0.

### 7.3. Experiment 3

For Experiment 3 we benchmarked the proposed branch and bound algorithm with (α,η)=(0.8,0.5%) against DFS and the greedy algorithm. The algorithm terminates after either the priority queue is empty or N=6000 iterations have completed. All algorithms are benchmarked with both uniform and nonuniform prior distributions.

**Figure 11:**
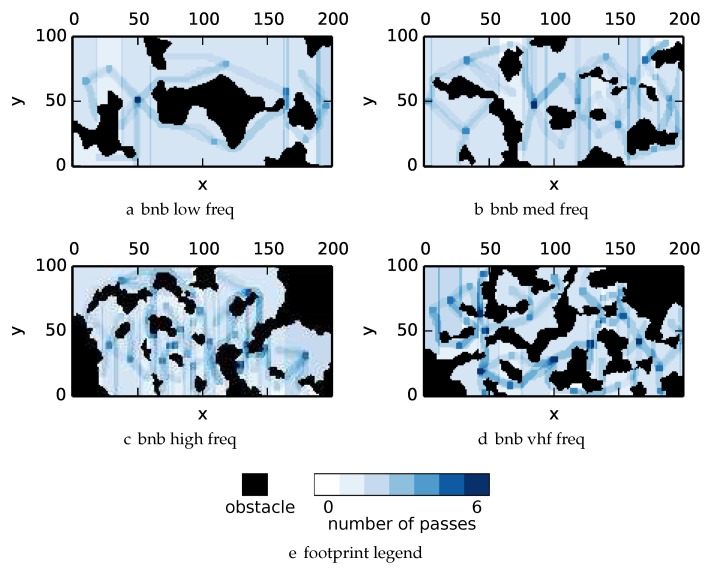
Multipass coverage plans in environments with uniform information for the ϵ-admissible B&B algorithm.

**Figure 12:**
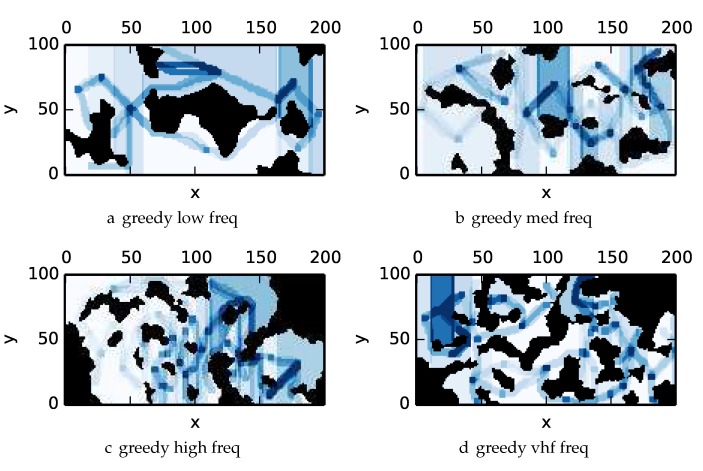
Multipass coverage plans in environments with uniform information for the greedy heuristic algorithm.

**Figure 13:**
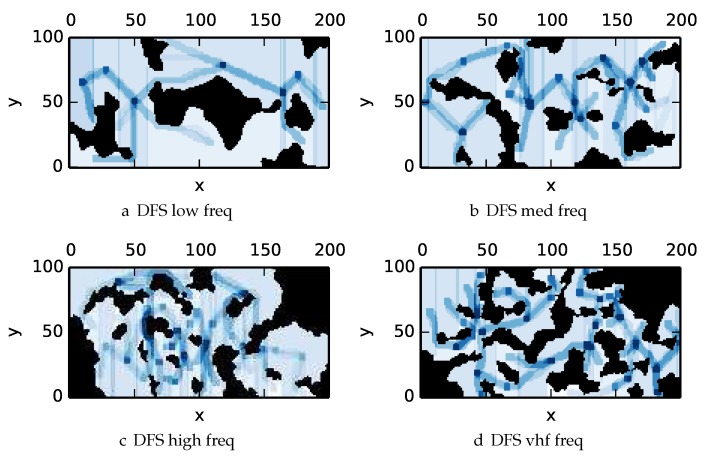
Multipass coverage plans in environments with uniform information for the DFS coverage planner algorithm.

**Figure 14:**
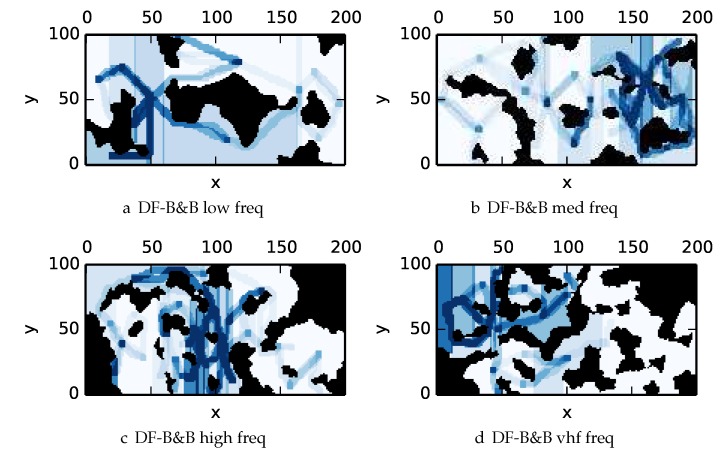
Multipass coverage plans in environments with uniform information for the DF-B&B coverage planner algorithm.

**Figure 15:**
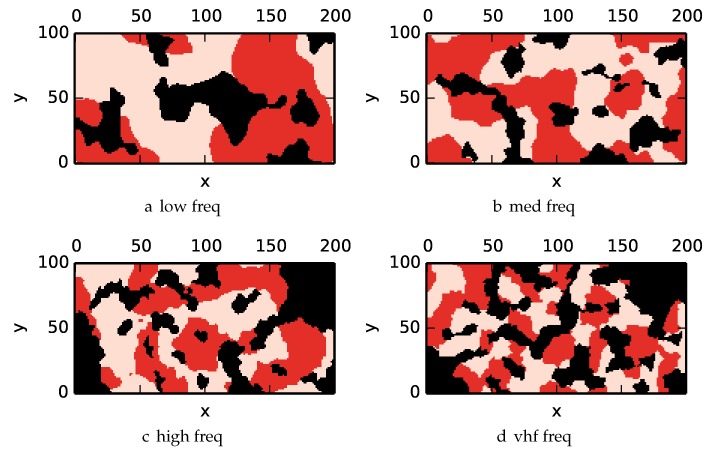
Example nonuniform environments showing obstacles (black), and the entropy of the cells before the search. The dark red indicates high entropy regions while the light red indicates low entropy regions.

**Figure 16:**
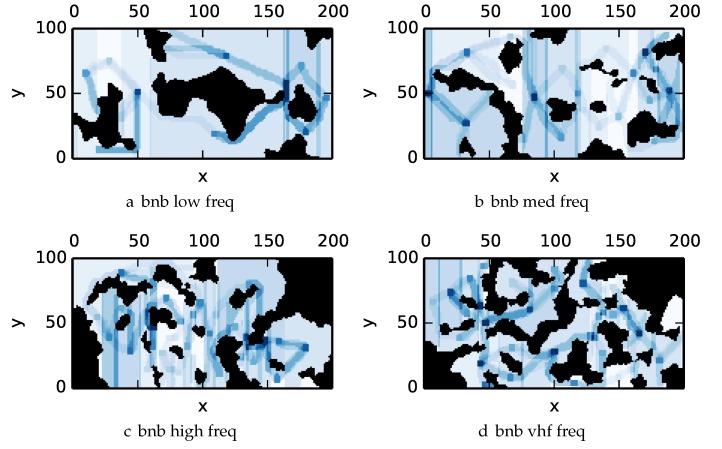
Multipass coverage plans in environments with nonuniform information for the ϵ-admissible B&B algorithm.

**Figure 17:**
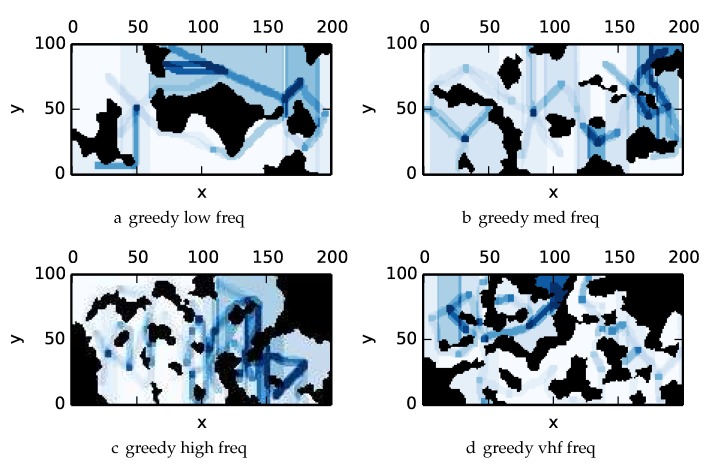
Multipass coverage plans in environments with nonuniform information for the greedy heuristic algorithm.

**Figure 18:**
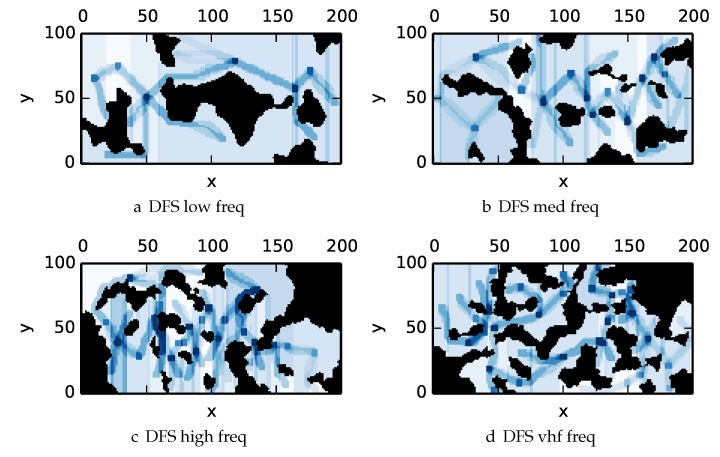
Multipass coverage plans in environments with nonuniform information for the DFS coverage planner algorithm.

**Figure 19:**
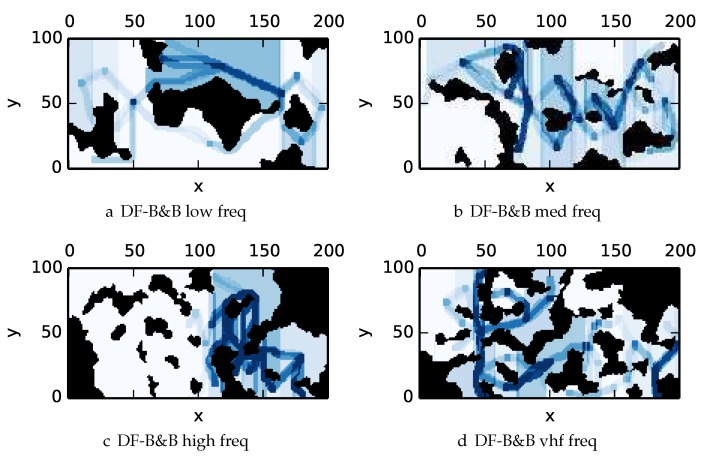
Multipass coverage plans in environments with nonuniform information for the DF-B&B coverage planner algorithm.

To get an understanding of how the various algorithms perform in different environments, we illustrate trajectory footprints representing median algorithm performance from ϵ-admissible B&B (Figure 11), greedy (Figure 12), DFS (Figure 13) and DF-B&B (Figure 14) in uniform environments. Figure 15 shows the entropy in example environments with information non-uniformly distributed in the environment. Figure 16, Figure 17, Figure 18 and Figure 19 show the median performance of their respective algorithms in nonuniform environments.

The trajectory footprints in Figure 11, Figure 12, Figure 13 and Figure 14 and Figure 16, Figure 17, Figure 18 and Figure 19 are an array representation of the best trajectory discovered by the mentioned algorithm, $\Phi_{T}\left[x,y\right]$. Areas not visited are white, obstacles are black, and blue regions indicate how many times the areas have been visited (see the legend in Figure 11e). Multipass coverage plans that are more uniform should be more uniform in color. Notice that all cells in a given region are not the same color; dots and lines shown within the regions show the information gathered between region traversals as these are visited additional times along commonly traveled paths between regions. Other lines are due to sensor footprints overlapping between adjacent regions.

**Figure 20:**
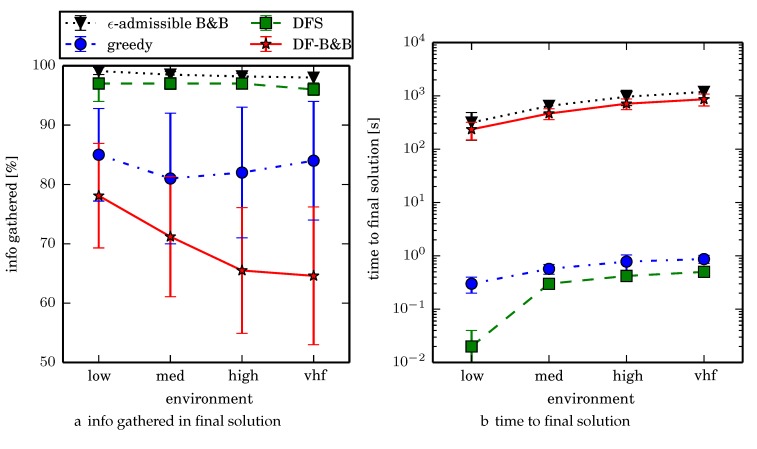

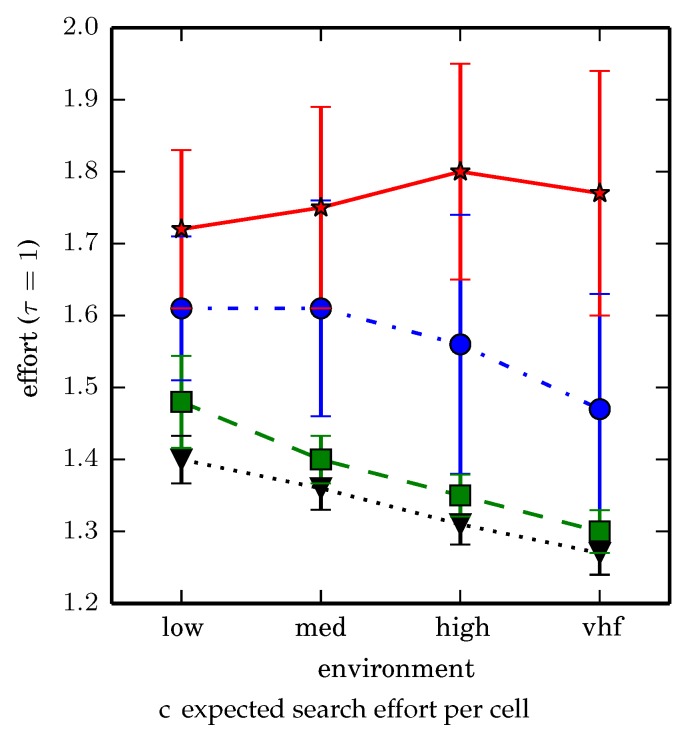
Results for Experiment 3 in uniform environment. See Table 2 for more detailed figures.

**Figure 21:**
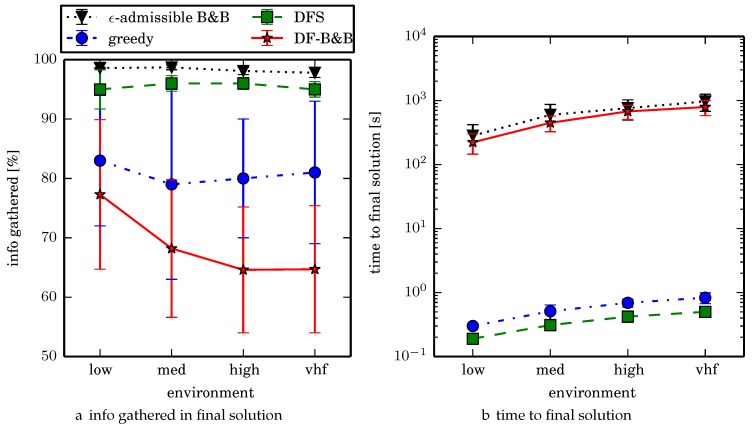

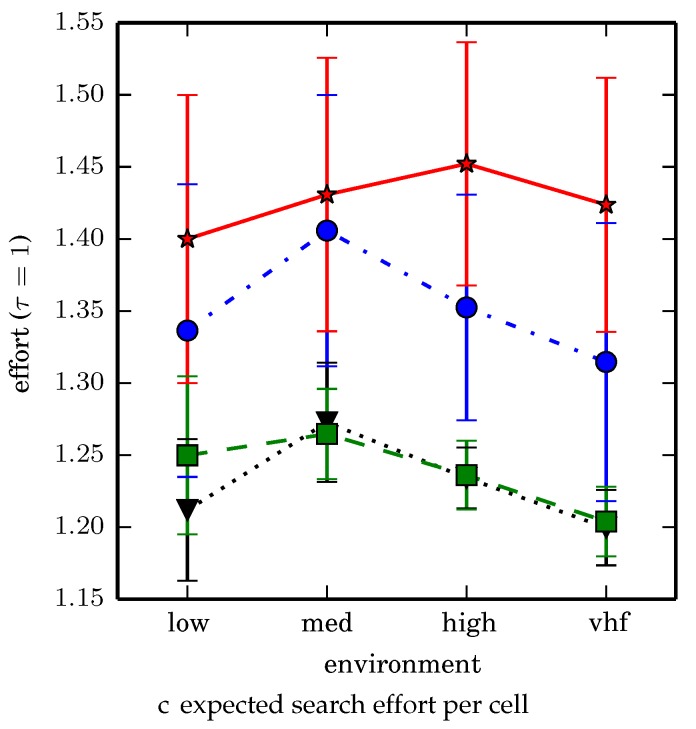
Results for Experiment 3 in nonuniform environment. See Table 3 for more detailed figures.

One can observe that branch and bound covers the environment most uniformly (resulting in the highest quality solution) while the greedy solver can get stuck or miss regions. DFS’s performance falls in between ϵ-admissible B&B and the greedy heuristic solver while DF-B&B has the worst performance. Results are summarized on the uniform environments in Table 2 and on the nonuniform environments in Table 3. $N_{sol}$ denotes the number of solution improvements found during the search. The described effort allocation model computes the expected time to search a cell in number of time constants (e.g., with τ=1), denoted as “effort” on Table 2 for the uniform environment using detection threshold $P_{neg}=1\%$. Figure 20 and Figure 21 summarize key results in Table 2 and Table 3 respectively.

## 8. Discussion

Experiment 1 identifies the parameters α,η that are to be used for ϵ-admissible B&B for the other experiments. The results of Experiment 2 demonstrate that ϵ-admissible B&B scales reasonably well with increasing environment size due to the hierarchical nature of the algorithm. Experimental results demonstrate that the ϵ-admissible heuristic is able to significantly speed up branch and bound with bounded loss in optimality.

We now direct our attention to assessing the four algorithms for their performances in solution quality and time to compute the solution in Experiment 3. ϵ-admissible B&B finds the highest quality solutions but takes the longest time to compute because it reasons over many candidate solutions. One limitation to ϵ-admissible B&B is that the heavy reliance on the iterative greedy heuristic and all the heuristic’s limitations (see Section 5.1) can cause the planner to continually expand too many candidate solutions at a fixed depth preventing the planner from finding a solution within the allotted time and memory constraints. Since Python is rather memory inefficient our experiments were limited to around 6000 iterations; more memory efficient languages ought to handle more iterations. Memory efficient search techniques such as beam-stack search [69] could alleviate such bottlenecks, but it is unknown how such techniques would interact with ϵ-admissible heuristics.

The heuristic solver DFS finds the second highest quality solutions in the shortest amount of time offering an effective trade-off in solution quality vs. speed to compute. We note that the quality of the DFS solutions degrades in environments with many more (smaller) regions due to the fact that more time is spent traversing between regions vs. searching the individual regions.

Comparing the two algorithms that gather the most information on average, ϵ-admissible B&B gathers 2.3% more information on average than DFS search taking 1–2 orders of magnitude longer to compute. To get a better sense of the real world performance improvement, ϵ-admissible B&B searches uniform environment at a rate that is 2.8% faster than the DFS coverage planner (where the search rate is inversely proportional to time to search a given cell), while ϵ-admissible B&B searches nonuniform environments at a rate that is 0.8% faster than DFS. We think that this decreased performance improvement in the nonuniform environments is a limitation of the Boustrophedon decomposition algorithm and/or the region coverage planner. Generating region coverage plans that factor in the information content by non-uniformly covering regions, or a polygonal decomposition that factors in information content (consider an extension of the weighted Voronoi Diagram that accounts for obstacles) would alleviate this and allow ϵ-admissible B&B to better reason about nonuniform distributions of information. When dealing with computational time budget constraints (fixed time budget for computing resources), we observe that ϵ-admissible B&B is an anytime algorithm. In the vhf uniform environments for example, around 90% of the time the first solution is discovered within 4.5 min (in contrast with about 90% of the trials terminating around 24 min).

One must consider the trade-offs between the added cost of computing better paths to the increased rate of rescuing survivors. We observe that the dramatic reduction in costs for supercomputing capabilities in recent years combined with the high value of a statistical life (around $7 million in 2005 USD [70]) imply that computer time is extremely cheap relative to human lives. Spending an additional $70,000 renting a sizeable chunk of supercomputing resources is considered cost effective if it increases the expected number of statistical lives rescued by at least 0.01 lives. Increasing the amount of area one is able to thoroughly search within 48 h by 2.8% is instrumental to increasing survival rates. More accurate figures are required for detailed analysis.

The greedy heuristic algorithm is able to find higher quality solutions than DF-B&B but not DFS or ϵ-admissible B&B. The greedy heuristic algorithm is also slower than DFS. The poor solution quality of DF-B&B despite the long time to compute the solution highlights the importance of generating effective heuristics to guide branch and bound to find high quality solutions. This is a finding that is not always recognized in the literature (usage of DF-B&B in place of heuristic guided B&B has occurred [11]). Note that DF-B&B is actually guaranteed to find optimal solutions when run to completion but is unable to find high quality solutions in a reasonable amount of time.

A practical way to verify the quality of heuristic solvers is through use of branch and bound which is guaranteed to find solutions near the optimal solution, suggesting use of branch and bound as a means of verifying suboptimal heuristic solvers.

## 9. Conclusions

In this paper we identify the task of generating multipass coverage plans for target search, as agents searching for targets must assess the trade-offs of searching a given region multiple times. This relatively unexplored area of research requires generating plans that visit most if not all of the workspace multiple times, where even seemingly small problems require long action sequences to solve.

Given the difficulty of the problem, we created the algorithm ϵ-admissible branch and bound (ϵ-admissible B&B) [13] which uses heuristics to improve the quality of the solution found in the given time. We benchmarked four different algorithms in Simplex environments assessing various performance metrics such as how long it takes to compute the solution and the quality of the solution (information gathered minimizing uncertainty in bits and expected time for a relief crew to search a cell).

The two most promising algorithms are ϵ-admissible B&B and the Depth First Search (DFS) heuristic solver [48]. ϵ-admissible B&B finds the highest quality solutions (gathers 2.2% more information than DFS and generates plans that search uniform environments 2.8% and nonuniform environments 0.8% faster than DFS) but takes the most time to compute. DFS on the other hand finds the second highest quality solutions but takes the shortest time to compute. The greedy algorithm and DF-B&B are both outperformed by DFS in terms of solution quality and time to solution. One of the greatest challenges to the practitioner not fully addressed by these findings is properly assessing the trade-offs in generating higher quality solutions at increased computational cost. Access to cheap supercomputing capabilities with the high value of a statistical life suggests that even small improvements in solution quality can be cost effective. However, for very large problem instances that we did not investigate in this paper (hundreds to thousands of regions, or tens of millions of cells), the findings of this study must be reinvestigated. Using branch and bound techniques may become intractable for very large problems, and heuristic techniques may offer the only feasible solution that can be computed in realistic time, with significant drops in solution quality.

For future work, we would like to improve the environment decomposition and region coverage planners in such a manner that can improve how information is gathered in a nonuniform environment. Extending techniques such as weighted Voronoi Diagrams offers a plausible means of doing so. We also plan to extend this work to a multiagent scenario by having multiple MAVs perform a coordinated search effort in a decentralized manner. During the plan execution, such agents will have to share their information in a robust fashion subject to communication network limitations. 

## Figures and Tables

**Table 1:** Subset of table of mutual information (info) of *q* future sensor measurements, indexed by previous number of sensor measurements equal to zero (*nz*0) and number of sensor measurements equal to one (*nz*1). Note that in the actual algorithm, the lookup table is computed for values *nz*0,*nz*1 ≤ 20 and q≤ 10.

*nz*0	0	1	2	0	1	2	0	1	2
*nz*1	0	0	0	1	1	1	2	2	2
**info (q= 1)**	0.390	0.209	0.050	0.209	0.390	0.209	0.050	0.209	0.390
**info (q= 2)**	0.599	0.347	0.094	0.347	0.599	0.347	0.094	0.347	0.599
**info (q= 3)**	0.737	0.432	0.125	0.432	0.737	0.432	0.125	0.432	0.737

**Table 2:** Experiment 3 results for uniform environments, mean of 80 trials.

**Env.**	**Succ. Rate**	**ϵ-Admissible Branch and Bound**					
		**1st Info**	**1st Time**	$N_{\mathrm{sol}}$	**Final Info**	**Final Time**	**Effort**
low	100%	92.8% ± 5.3%	5.8 ± 9	10 ± 4.1	99.1% ± 0.6%	316 ± 169	1.4 ± 0.033
med	98.8%	95.9% ± 0.2%	27 ± 47	9.3 ± 4.5	98.5% ± 0.5%	648 ± 175	1.36 ± 0.030
high	93.8%	96.8% ± 1.1%	76 ± 77	7.4 ± 4.2	98.2% ± 0.5%	961 ± 308	1.31 ± 0.028
vhf	98.8%	96.6% ± 0.9%	129 ± 143	8.9 ± 4.7	98.0% ± 0.9%	1185 ± 273	1.27 ± 0.030
**Env.**		**Greedy**			**DFS Coverage Planner**		
		**Info**	**Time**	**Effort**	**Info**	**Time**	**Effort**
low		85% ± 7.8%	0.3 ± 0.1	1.61 ± 0.01	97% ± 3%	0.2 ± 0.02	1.48 ± 0.064
med		81% ± 11%	0.57± 0.12	1.61 ± 0.15	97% ± 1.0%	0.3 ± 0.03	1.40 ± 0.033
high		82% ± 11%	0.78 ± 0.26	1.56 ± 0.18	97% ± 0.7%	0.42 ± 0.04	1.35 ± 0.029
vhf		84% ± 10%	0.87 ± 0.15	1.47 ± 0.16	96% ± 1%	0.5 ± 0.04	1.30 ± 0.18
**Env.**		**Depth First Branch and Bound**					
		**1st Info**	**1st Time**	$N_{\mathrm{sol}}$	**Final Info**	**Final Time**	**Effort**
low		71.2% ± 10%	8.5 ± 4.7	42 ± 17	78.1% ± 8.8%	235 ± 85	1.72 ± 0.11
med		66.3% ± 11.4%	31 ± 12	38 ± 16	71.2% ± 10.1%	466 ± 107	1.75 ± 0.14
high		61.9% ± 11.5%	69 ± 21	34 ± 15	65.5% ± 10.6%	712 ± 160	1.80 ± 0.15
vhf		61.6% ± 11.8%	107 ± 31	30 ± 15	64.6% ± 11.6%	862 ± 216	1.77 ± 0.17

**Table 3:** Experiment results for nonuniform environments, mean of 80 trials.

**Env.**	**Succ. Rate**	**ϵ-Admissible Branch and Bound**					
		**1st Info**	**1st Time**	**$N_{\mathrm{sol}}$**	**Final Info**	**Final Time**	**Effort**
low	100%	93.2% ± 4.3%	6.8 ± 20	11 ± 5.8	98.6% ± 0.1%	282 ± 137	1.21 ± 0.05
med	98.8%	97% ± 0.4%	31 ± 51	9.5 ± 4.3	98.7% ± 0.4%	596 ± 271	1.27 ± 0.04
high	100%	96.6% ± 1.5%	111 ± 141	7.5 ± 4.1	98.1% ± 0.6%	760 ± 264	1.23 ± 0.02
vhf	95%	96.3% ± 1.2%	151 ± 199	8.7 ± 5.1	97.8% ± 0.8%	965 ± 288	1.20 ± 0.03
**Env.**		**Greedy**			**DFS Coverage Planner**		
		**Info**	**Time**	**Effort**	**Info**	**Time**	**Effort**
low		83% ± 11%	0.3 ± 0.01	1.34 ± 0.10	95% ± 3.3%	0.19 ± 0.03	1.25 ± 0.05
med		79% ± 16%	0.51 ± 0.13	1.41 ± 0.09	96% ± 1.3%	0.31 ± 0.03	1.26 ± 0.03
high		80% ± 10%	0.69 ± 0.01	1.35 ± 0.08	96% ± 1.0%	0.42 ± 0.04	1.24 ± 0.02
vhf		81% ± 12%	0.83 ± 0.16	1.31 ± 0.10	95% ± 1.3%	0.5 ± 0.04	1.20 ± 0.02
**Env.**		**Depth First Branch and Bound**					
		**1st Info**	**1st Time**	$N_{\mathrm{sol}}$	**Final Info**	**Final Time**	**Effort**
low		70.2% ± 13.9%	7.3 ± 4.4	36 ± 16	77.3% ± 12.6%	222 ± 78	1.4 ± 0.1
med		62.6% ± 13.0%	29 ± 12	38 ± 16	68.2% ± 11.6%	448 ± 124	1.43 ± 0.09
high		60.9% ± 11.2%	68 ± 21	30 ± 14	64.6% ± 10.6%	675 ± 169	1.45 ± 0.08
vhf		61.3% ± 11.4%	101 ± 31	28 ± 10	64.7% ± 10.7%	790 ± 210	1.42 ± 0.09
